# Development of Fluorescent Turn-On Probes for CAG-RNA Repeats

**DOI:** 10.3390/bios12121080

**Published:** 2022-11-25

**Authors:** Matthew Ho Yan Lau, Chun-Ho Wong, Ho Yin Edwin Chan, Ho Yu Au-Yeung

**Affiliations:** 1 Department of Chemistry, The University of Hong Kong, Pokfulam Road, Hong Kong, China; 2 School of Life Sciences, The Chinese University of Hong Kong, Hong Kong, China; 3 Gerald Choa Neuroscience Centre, The Chinese University of Hong Kong, Hong Kong, China; 4 Nexus of Rare Neurodegenerative Diseases, School of Life Sciences, The Chinese University of Hong Kong, Hong Kong, China; 5 State Key Laboratory of Synthetic Chemistry, The University of Hong Kong, Pokfulam Road, Hong Kong, China

**Keywords:** fluorescent probe, AIE, RNA, CAG repeats

## Abstract

Fluorescent sensing of nucleic acids is a highly sensitive and efficient bioanalytical method for their study in cellular processes, detection and diagnosis in related diseases. However, the design of small molecule fluorescent probes for the selective binding and detection of RNA of a specific sequence is very challenging because of their diverse, dynamic, and flexible structures. By modifying a bis(amidinium)-based small molecular binder that is known to selectively target RNA with CAG repeats using an environment-sensitive fluorophore, a turn-on fluorescent probe featuring aggregation-induced emission (AIE) is successfully developed in this proof-of-concept study. The probe (**DB-TPE**) exhibits a strong, 19-fold fluorescence enhancement upon binding to a short CAG RNA, and the binding and fluorescence response was found to be specific to the overall RNA secondary structure with A·A mismatches. These promising analytical performances suggest that the probe could be applied in pathological studies, disease progression monitoring, as well as diagnosis of related neurodegenerative diseases due to expanded CAG RNA repeats.

## 1. Introduction

Polyglutamine (PolyQ) diseases, including Huntington’s disease, spinobulbar muscular atrophy and spinocerebellar ataxias, are a group of inheritable neurodegenerative disorders characterized by the expansion of a CAG trinucleotide repeat of the affected genes and the expression of polyQ proteins with a glutamine-enriched tract, in which the number of CAG repeats in patients or carriers can reach from 40 to over 100 as compared to that of 35–50 in healthy individuals [1,2,3,4,5]. The polyQ proteins are neurotoxic and can cause a wide range of cellular dysfunctions in neurons. Taking Huntington’s disease as an example, the mutant *HTT* gene encodes the toxic and aggregation-prone mutant huntingtin (mHTT) protein with an expanded polyQ tract translated from the expanded CAG repeats, and the accumulation of the mHTT protein will lead to neuronal cell death due to altered neural circuitry, mitochondrial dysfunction, transcriptional dysregulation, disrupted protein homeostasis, impaired protein degradation, and aberrant activation of stress responses [6,7,8,9,10]. In addition to the toxicity due to the misfolding, accumulation, and aggregation of the polyQ proteins, recent studies are showing that the expanded CAG RNA repeats are also neurotoxic [11,12,13,14]. A recent work has demonstrated that cellular expression of short CAG (sCAG) RNAs can hybridize with and silence the CUG-containing mRNA in the *Nudix hydrolase 16* (*NUDT16*) gene and lead to DNA damages and cellular apoptosis [15]. Small molecule inhibitors that selectively bind to the CAG RNA repeats are therefore implicated in not only the development of therapeutic strategies for treating polyQ diseases, but also their rapid detection and diagnosis.

**Figure 1:**
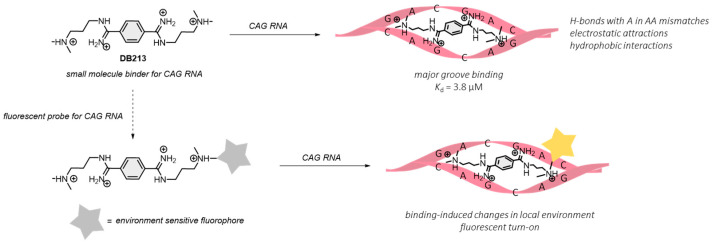
Chemical structure of **DB213** that binds selectively to CAG RNA, and the design of a fluorescent probe for CAG RNA.

Designing small molecule binders and related fluorescent probes for specific RNA sequences is, however, very challenging because of the highly diverse, dynamic and flexible structures of RNA [16,17,18]. Recently, we have identified the bis(amidinium) compound, **DB213**, as a small molecule drug candidate for treating polyQ RNA toxicity. Detail binding and NMR studies showed that **DB213** binds with a *K*_d_ of 3.8 µM to the major groove of an sCAG hairpin (*rGG-(CAG)_6_-CC*) and interacts specifically with two consecutive A·A mismatches via a combination of hydrogen bonds, electrostatic, and other interactions (Figure 1) [15]. The binding is selective, and a much weaker binding was observed for CUG RNA [19,20,21] and the rCAG/CUG heteroduplex formed from *rGG-(CAG)_4_-(CUG)_2_-CC* (*K*_d_ = 19.2 µM) [15]. Further cellular studies and biochemical analysis also showed that **DB213** did not affect the transcription level of the mRNA of a CAG repeat disease gene, and that it could suppress toxicity due to CAG RNA but not polyQ proteins, showing that **DB213** is specific to the CAG RNA over the corresponding DNA and proteins [15]. With these promising binding properties, we propose that **DB213** could be turned into a fluorescent probe for CAG RNA repeats by appending an environment sensitive fluorophore on the bis(amidinium) core, such that the probe would elicit a fluorescent response upon binding to the target CAG RNA as a result of a change in the local environment. In this work, we present our results on this proof-of-concept study including the design, synthesis and solution characterizations of three fluorophore-appended **DB213** derivatives as potential fluorescent probes for CAG RNA. In particular, **DB-TPE** which features a tetraphenylethylene fluorophore was found to exhibit a strong fluorescence enhancement upon binding to CAG RNA via aggregation-induced emission (AIE). 

## 2. Materials and Methods

All reagents were purchased from commercial suppliers (Aldrich, Dkmchem, Energy, J & K) and used without further purification unless otherwise specified. All solvents were of analytical grade (ACL Labscan, DUKSAN Pure Chemicals). Dried solvents were distilled over CaH_2_ (CHCl_3_, EtOH, MeOH). Thin layer chromatography was performed on silica gel 60 F254 (Merck) and column chromatography was carried out using silica gel 60F (Silicycle), neutral or basic aluminium oxide Brockmann I (Acros Organics). Detailed synthetic procedures and characterization of all other starting materials are described in the Supplementary File S1. ^1^H and ^13^C{1H} NMR spectra were obtained from a Brucker DPX 400 or Brucker DPX 500 spectrometer and signals were referenced to solvent residues. LC-MS analyses were performed on a UPLC-MS system with a Waters UPLC coupled to a 2489 UV/Vis detector and an ACQUITY QDa MS detector. High resolution ESI-MS data was obtained from a Waters Micromass Premier Q-ToF tandem mass spectrometer.

Synthesis of **DB-Res** was carried out as follows. Under an argon atmosphere, a mixture of **DB-N_3_** (10 mg, 0.03 mmol), **Res-alkyne** (8 mg, 0.03 mmol), and [Cu(CH_3_CN)_4_](PF_6_) (1 mg, 0.003 mmol) in DMSO (3 mL) was stirred at room temperature for overnight. The resulting mixture was purified by a neutral alumina column with CH_2_Cl_2_/MeOH (*v*/*v* = 8:2) as the eluent. Fractions containing the product was combined and concentrated using a rotary evaporator, to which a saturated HCl solution in dry EtOH (1 mL) was added. The mixture was stirred at room temperature for 3 h and centrifuged (4000 rpm, 3 min). The solution was decanted and the remaining solid was washed with dry EtOH (10 mL × 1) and dry diethyl ether (10 mL × 3). The orange-red solid obtained was dried under vacuum. Yield = 15 mg, 73%. ^1^H NMR (500 MHz, CD_3_OD, 298 K): δ 8.27 (s, 1H), 7.99–7.95 (m, 2H), 7.95–7.91 (m, 2H), 7.78 (d, *J* = 8.9 Hz, 1H,), 7.54 (d, *J* = 9.8 Hz, 1H), 7.17 (d, *J* = 2.6 Hz, 1H), 7.13 (dd, *J* = 8.9, 2.7 Hz, 1H), 6.85 (dd, *J* = 9.8, 2.1 Hz, 1H), 6.28 (d, *J* = 2.1 Hz, 1H), 5.35 (s, 2H), 4.62 (t, *J* = 6.0 Hz, 2H), 3.57 (t, *J* = 6.7 Hz, 2H), 3.26 (t, *J* = 6.7 Hz, 2H), 2.96 (t, *J* = 6.0 Hz, 2H), 2.84–2.78 (m, 2H), 2.56 (s, 9H), 2.38–2.34 (m, 2H), 2.06 (quint, *J* = 7.2 Hz, 2H), and 1.85 (quint, *J* = 6.8 Hz, 2H). ^13^C{^1^H} NMR (125 MHz, CD_3_OD, 298 K): δ 188.3, 164.9, 164.6, 164.2, 151.9, 147.1, 146.6, 143.9, 136.6, 135.0, 134.8, 134.7, 133.0, 130.1, 130.0, 130.0, 126.4, 115.7, 106.9, 102.3, 63.3, 57.2, 56.7, 55.1, 44.6, 44.5, 42.3, 42.3, 42.2, 26.2, and 25.5. HR-ESI-MS (+ve) calculated for C_34_H_42_N_10_O_3_ [M+H]^+^ (*m*/*z*): 639.3514, found: 639.3504.

Synthesis of **DB-Dan** was carried out as follows. Under an argon atmosphere, a mixture of **DB-N_3_** (43 mg, 0.11 mmol), **Dan-alkyne** (35 mg, 0.12 mmol), and [Cu(CH_3_CN)_4_](PF_6_) (4 mg, 0.01 mmol) in MeCN/MeOH (2 mL/0.5 mL, *v*/*v* = 8:2) was stirred at room temperature for overnight. Solvents were removed using a rotary evaporator, and the residue was purified by a basic alumina column using a gradient mixture of CH_2_Cl_2_/MeOH (*v*/*v* = 9:1 to 8:2) as the eluent. Fractions containing the product were combined and concentrated using a rotary evaporator, to which a saturated HCl solution in dry ethanol (1 mL) was added. The mixture was heated at 60 °C for 3 h, cooled to room temperature, and centrifuged (4000 rpm, 3 min). The solution was decanted and the remaining solid was washed with dry ethanol (10 mL × 1) and dry diethyl ether (10 mL × 3). The resulting solid was dried under vacuum to obtain the product as a pale-yellow solid. Yield = 20 mg, 21%. ^1^H NMR (400 MHz, CD_3_OD, 298 K): δ 8.52 (d, *J* = 8.4 Hz, 1H), 8.32 (d, *J* = 8.7 Hz, 1H), 8.15 (d, *J* = 7.2 Hz, 1H), 7.71 (s, 4H), 7.60–7.48 (m, 3H), 7.25 (d, *J* = 7.5 Hz, 1H), 4.31 (t, *J* = 6.1 Hz, 2H), 4.11 (s, 2H), 3.33 (t, *J* = 7.1 Hz, 2H), 3.18 (t, *J* = 6.7 Hz, 2H), 2.87 (s, 6H), 2.74 (t, *J* = 6.2 Hz, 2H), 2.54–2.38 (m, 4H), 2.27 (s, 6H), 2.25 (s, 3H), 1.87 (quint, *J* = 7.2 Hz, 2H), and 1.75 (quint, *J* = 6.9 Hz, 2H). ^13^C{^1^H} NMR (125 MHz, CD_3_OD, 298 K): δ 163.8, 157.3, 153.1, 146.0, 139.0, 138.6, 137.2, 131.1, 131.0, 130.9, 130.2, 129.1, 128.5, 128.4, 124.8, 124.3, 120.6, 116.3, 58.2, 57.6, 56.0, 45.8, 45.4, 42.9, 42.5, 42.2, 39.2, 27.6, and 27.3. HR-ESI-MS (+ve) calculated for C_34_H_49_N_11_O_2_S [M+H]^+^ (*m*/*z*): 676.3864, found: 676.3870.

Synthesis of **DB-TPE** was carried out as follows. Under an argon atmosphere, a mixture of **DB-alkyne** (18 mg, 0.05 mmol), **TPE-N_3_** (24 mg, 0.06 mmol), and [Cu(CH_3_CN)_4_](PF_6_) (2 mg, 0.005 mmol) in MeCN/MeOH/CH_2_Cl_2_ (2 mL/1 mL/1 mL, *v*/*v* = 2:1:1) was stirred at room temperature for overnight. The solvents were removed using a rotary evaporator, and the residue was washed with dry diethyl ether (10 mL × 2). A saturated HCl solution in dry ethanol (1 mL) was added to the residue, and the mixture was heated at 60 °C for 3 h, cooled to room temperature, and centrifuged (4000 rpm, 3 min). The solution was decanted and the remaining solid was washed with dry ethanol (10 mL × 1) and dry diethyl ether (10 mL × 3). The resulting solid was dried under vacuum to a obtain the product as a yellow solid. Yield = 42 mg, 93%. ^1^H NMR (400 MHz, CD_3_OD, 298 K): δ 8.39 (s, 1H), 8.03 (s, 4H), 7.16–7.01 (m, 12H), 7.01–6.88 (m, 7H), 5.58 (s, 2H), 4.59–4.37 (m, 2H), 3.72–3.55 (m, 2H), 3.53–3.39 (m, 4H), 2.94 (s, 6H), 2.90 (s, 3H), 2.39–2.28 (m, 2H), and 2.28–2.18 (m, 2H). ^13^C{^1^H} NMR (125 MHz, CD_3_OD, 298 K): δ 165.1, 144.8, 144.7, 143.1, 141.5, 134.8, 132.9, 132.3, 132.2, 130.3, 128.9, 128.8, 128.7, 127.7, 127.7, 56.5, 43.9, 41.7, and 24.2. HR-ESI-MS (+ve) calculated for C_47_H_53_N_9_ [M+H]^+^ (*m*/*z*): 744.4497, found: 744.4494.

Fluorescence spectra were recorded on an Edinburgh Instruments FS5 Spectrofluorometer equipped with a 150 W CW ozone-free xenon arc lamp and a Photomultiplier R928P detection unit with spectral coverage of 200–870 nm. Fluorescence studies were performed in quartz cuvettes of 1 cm path length and 1.5 mL cell volume, and double distilled water was used in all measurement. Stock solutions of **DB-Res**, **DB-Dan**, and **DB-TPE** in water were diluted to 10 μM with H_2_O and/or THF to the required THF/H_2_O ratios. **DB-Res**, **DB-Dan**, and **DB-TPE** were excited at 475 nm, 330 nm, and 300 nm, respectively. Synthetic RNAs, *rGG-(CAG)_6_-CC* (*5′-rGG CAG CAG CAG CAG CAG CAG CC-3′*), *rGG-(CAG)_3_-CC* (*5′-rGG CAG CAG CAG CC-3′*), and *rGG-(CUG)_3_-CC* (*5′-rGG CUG CUG CUG CC-3′*) were purchased from Integrated DNA Technologies, and stock solutions at 100 μM were prepared by dissolving the RNA in UltraPure™ DNase/RNase-free distilled water. For measurements in the presence of RNA, RNA sample were diluted with water, heated at 95 °C for 2 min, and slowly cooled to room temperature and added to a sample solution containing 10 μM of the fluorescent probe in 20 mM MOPS buffer (pH 7.0) in the presence of 300 mM NaCl.

Dissociation constant (*K_d_*) of **DB-TPE** towards *rGG-(CAG)_6_-CC* was determined by non-linear curve fitting of the fluorescence data using the following equation:

$$F_{R}=F_{0}+\frac{F_{MAX}-F_{0}}{2{\left[P\right]}_{0}}\times \left[\left({\left[P\right]}_{0}+\left[R\right]+K_{d}\right)-\sqrt{{\left({\left[P\right]}_{0}+\left[R\right]+K_{d}\right)}^{2}-4{\left[P\right]}_{0}\left[R\right]}\right]$$

where $F_{R}$ is the fluorescence intensity at 463 nm for a given concentration of RNA $\left[R\right]$, $F_{0}$ is the fluorescence intensity in the absence of RNA, $F_{MAX}$ is the maximum fluorescence intensity at saturation, ${\left[P\right]}_{0}$ is the total concentration of **DB-TPE** (10 μM), and $\left[R\right]$ is the total concentration of RNA.

## 3. Results and Discussions

**Figure 2:**
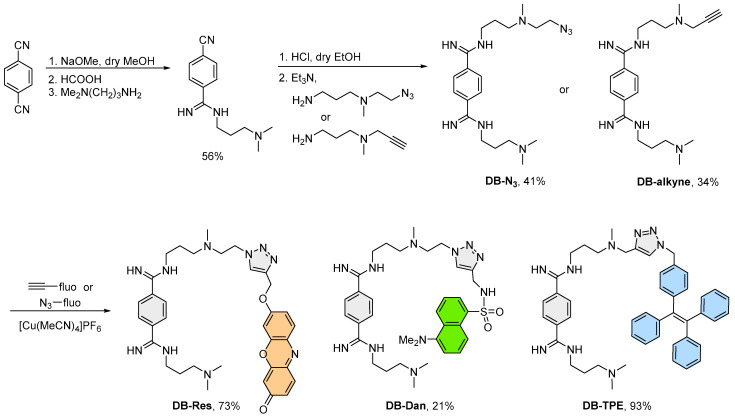
Synthesis of **DB-Res**, **DB-Dan**, and **DB-TPE**.

Three bis(amidinium) derivatives have been designed and synthesized as potential fluorescent probes for CAG RNA based on the structure of **DB213** (Figure 2). In particular, three different fluorophores, namely resorufin, dansyl, and tetraphenylethylene (TPE), were chosen as the fluorescent reporters that are sensitive to the local environment for obtaining a fluorescence response upon RNA binding. The probes were synthesized via a copper-catalyzed azide–alkyne cycloaddition between **DB-N_3_** or **DB-alkyne** and the corresponding fluorophore-containing click partners. Of note, compared to oligonucleotide-based probes that can engage in sequence-specific hybridization with the target DNA or RNA [22,23], small molecule probes are more versatile with diverse structure, and their photophysical, chemical, and biological properties can be readily tuned via chemical modifications [24,25]. However, due to the highly diverse and dynamic structures of RNA, small molecular probes that can selectively recognize a specific RNA sequence are scarce [16,17,18]. In addition to RNA binding, an appropriate mechanism to induce a change in the fluorescent properties upon binding is also essential for a successful RNA probe. While the resorufin and dansyl fluorophores are known to be sensitive to the local environment and could elicit a change in their emission due to a different degree of the intramolecular charge transfer (ICT) [26,27,28,29], TPE is a representative AIEgen that the restricted intramolecular motions upon binding will block the non-radiative decay pathway and result in an emission turn-on [30]. 

**Figure 3:**
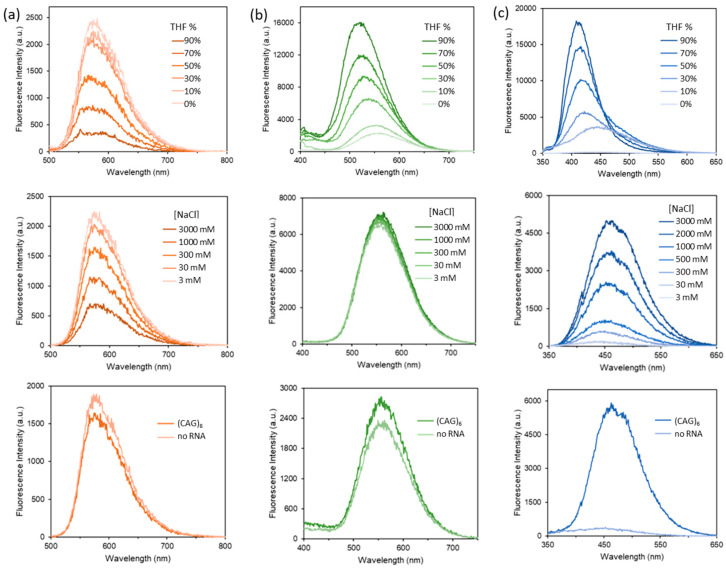
Fluorescence spectra of 10 µM (**a**) **DB-Res** (λ_ex_ = 475 nm), (**b**) **DB-Dan** (λ_ex_ = 330 nm), and (**c**) **DB-TPE** (λ_ex_ = 300 nm) in different water/THF ratios (upper panel), ionic strengths with various NaCl concentrations (middle panel), and the presence of 1 eq. of a (CAG)_6_ RNA (lower panel).

All the three probes have been characterized by ^1^H NMR, ^13^C NMR, HR-ESI-MS, and UV–Vis. Fluorescent properties of the probes in response to environment changes were evaluated to assess their applicability in fluorescent RNA sensing. Figure 3 shows the emission spectra of 10 µM solutions of **DB-Res**, **DB-Dan**, and **DB-TPE** in water/THF mixtures of various ratios and ionic strengths (from 3 mM to 3 M NaCl). For **DB-Res**, weaker fluorescence was observed when there is an increase in the percentage of THF, and a 7.5-fold decrease in the emission intensity at 574 nm was found at 90% THF when compared to that at pure water. The weaker emission can be explained by the formation of non-emissive aggregates of the probe when the solvent becomes more non-polar. Consistent with this, weaker fluorescence was also observed when solvent ionic strength increased, and there was a 3.2-fold decrease in fluorescence in the presence of 3 M NaCl. On the other hand, an increase in fluorescence, along with a blue shift of the emission maximum, was observed for **DB-Dan** upon increasing the THF percentage. At 90% THF, a 6.8-fold emission enhancement and a blue shift of the emission maximum by 41 nm (from 562 nm to 521 nm) was resulted, and these solvatochromic and fluorogenic effects are consistent with an ICT mechanism [31,32]. No significant change in the emission maximum and intensity was found for **DB-Dan** at different ionic strengths. Consistent with the AIE effect of TPE, a 10 µM solution of the aqueous soluble, cationic **DB-TPE** in water was weakly emissive and can be ascribed to the rapid non-radiative decay due to intramolecular rotations of the phenyl units [33,34]. Upon increasing the THF content to 90%, a strong emission enhancement by 85-fold and a blue shift in the emission maximum by 42 nm were observed. The decrease in solubility of **DB-TPE** upon addition of THF likely resulted in the aggregation of the probe and restricted the intramolecular rotations that activated the TPE emission. Compared to the emission intensity in the presence of 3 mM NaCl, a 24-fold increase in fluorescence was observed in the presence of 3 M NaCl, showing that the salting-out effect at high ionic strength can also trigger the AIE.

Fluorescence response of the probes was further tested using a synthetic RNA with the sequence *rGG-(CAG)_6_-CC* RNA as a model of CAG repeats. Previous studies have shown that this particular sCAG RNA will form a hairpin structure in solution with which **DB213** can selectively bind with a strong affinity (*K*_d_ = 3.8 µM) [15,35]. Fluorescence response to the RNA was studied with 10 µM of the probe in the presence of 300 mM NaCl in 20 mM MOPS buffer at pH 7. As shown in Figure 3, only a minimal change in the emission was observed in the presence of 1 eq. of the (CAG)_6_ RNA for **DB-Res** and **DB-Dan**, suggesting that the change in the local environment upon binding may not be significant enough to result in an obvious fluorescent response. On the contrary, a 19-fold emission enhancement of **DB-TPE** was observed under the same condition, suggesting that the AIE probe has a promising analytical potential for CAG RNA.

**Figure 4:**
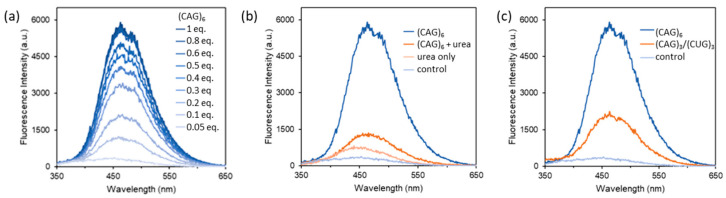
Fluorescence spectra of 10 µM **DB-TPE** (λ_ex_ = 300 nm) in the presence of (**a**) increasing amount of (CAG)_6_ RNA from 0.05 to 1 eq., (**b**) 1 eq. of (CAG)_6_ RNA denatured by 1 M urea, and (**c**) 1 eq. of complementary (CAG)_3_/(CUG)_3_ RNA duplex.

Response of **DB-TPE** towards the (CAG)_6_ RNA was studied in more details. Figure 4 shows the concentration dependent response of the probe, and the relative emission intensity at 463 nm was found to increase from 3.5-fold to 18.6-fold when the amount of (CAG)_6_ RNA increased from 0.05 eq. to 1 eq. Binding strength of **DB-TPE** to the (CAG)_6_ RNA was obtained from the fluorescence titration data, and a *K*_d_ of 2 µM was found by fitting the binding isotherm to a 1:1 binding model. The *K*_d_ of **DB-TPE** is comparable to that of the binding of **DB213** to the same (CAG)_6_ RNA, suggesting that both bis(amidinium) compounds share a similar binding mode that targets the major groove and overall secondary structure of the sCAG RNA with A·A mismatches in the hairpin structure. Consistent with this, a much weaker fluorescence, likely due to the non-specific electrostatic interactions between the cationic bis(amidinium) and anionic nucleic acid, was observed when the sCAG RNA was denatured by urea (Figure 4b), or in the presence of 1 eq. of the canonically-paired duplex consisting of *rGG-(CAG)_3_-CC* and *rGG-(CUG)_3_-CC* with complementary A·U pairs (Figure 4c). Overall, these results clearly demonstrate the favorable analytical performance of **DB-TPE** as a turn-on fluorescent probe for the selective sensing of RNA with CAG repeats that contain A·A mismatches.

## 4. Conclusions

In summary, a fluorescent turn-on probe that selectively binds to sCAG RNA with a strong fluorescence enhancement is successfully developed. Despite the various challenges in specific RNA recognition due to their dynamic structures, by using tetraphenylethylene as an AIE-active fluorophore to decorate **DB213**, a known small molecule inhibitor that selectively binds to the major groove of CAG RNA with A·A mismatches, **DB-TPE** was obtained and found to display a strong (~19-fold) fluorescence enhancement upon binding to sCAG RNA with an apparent *K*_d_ of 2 µM. Comparative fluorescence studies showed that **DB-TPE** binds and reports the presence of sCAG RNA with a specific secondary structure. With this promising analytical performance, **DB-TPE** not only could be employed in the sensitive detection of CAG RNA for diagnostic applications of CAG repeats-related neurodegenerative diseases, but may also allow for the real-time monitoring of the CAG RNA for further understanding of the disease in cell models. Further works on applying the probe in biological samples and specimens are currently underway in our laboratory. 

## Data Availability

The data presented in this study are available in supplementary material.

## Supplementary Materials

The following supporting information can be downloaded at https://www.mdpi.com/article/10.3390/bios12121080/s1, File S1: synthetic procedures of precursors, NMR, MS and UV-Vis spectra. References [36,37] are citied in the Supplementary Materials.
