# Supplementary material for: Development of Fluorescent Turn-On Probes for CAG-RNA Repeats
Source: Biosensors (Basel). 2022 Nov 25;12(12):1080. doi: 10.3390/bios12121080 (PMC9775061; doi:10.3390/bios12121080)
Supplement: Supplementary file 1 [file biosensors-12-01080-s001.zip › biosensors-2051279-supplementary.pdf]

Supporting Information for

Development of Fluorescent Turn-on Probes for CAG-RNA Repeats

Matthew Ho Yan Lau, Chun-Ho Wong, Ho Yin Edwin Chan and Ho Yu Au-Yeung

All reagents were purchased from commercial suppliers (Aldrich, Dkmchem, Energy and J & K) and used without further purification unless otherwise specified. All solvents were of analytical grade (ACI Labscan and DUKSAN Pure Chemicals). Dry solvents were distilled under argon atmosphere from sodium-benzophenone ketyl still pots (Et_2O and THF) or over CaH_2 ($CHCl_3$, EtOH, and MeOH). Thin layer chromatography (TLC) was performed on silica gel 60 F254 (Merck, Germany, Aluminium sheet). Column chromatography was carried out using silica gel 60F (Silicycle), neutral aluminium oxide Brockmann I (Acros Organics), and basic aluminium oxide Brockmann I (Acros Organics). **TPE-N_3** and **Dan-alkyne** were synthesized according to literature procedures.[1,2]

^{1}H and ^{13}C{1H} NMR spectra were obtained from a Brucker DPX 400 or Brucker DPX 500 spectrometer. NMR signals were referenced to solvent residues. LC-MS analyses were performed on a UPLC-MS system with a Waters UPLC coupled to a 2489 UV/Vis detector and an ACQUITY QDa MS detector. High resolution ESI-MS data was obtained from a Waters Micromass Premier Q-ToF tandem mass spectrometer. UV-Vis spectra were recorded on a Hitachi UH5300 UV-Vis spectrophotometer. Fluorescence spectra were recorded on an Edinburgh Instruments FS5 Spectrofluorometer equipped with a 150 W CW ozone-free xenon arc lamp and a Photomultiplier R928P detection unit with spectral coverage of 200 nm – 870 nm.

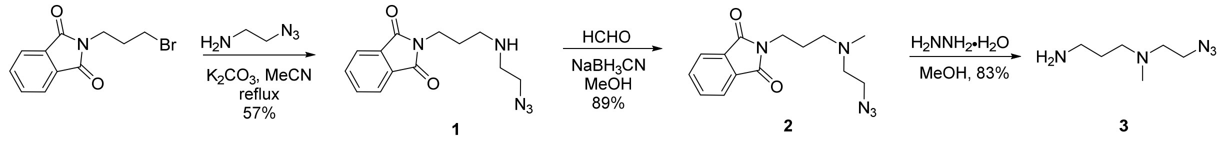

Synthesis of **1**. A mixture of *N*-(3-bromopropyl)phthalimide (1.25 g, 4.67 mmol), $N_3CH_2CH_2NH_2$ (0.60 g, 7.00 mmol), and potassium carbonate (1.93 g, 14.0 mmol) was refluxed in MeCN (150 mL) overnight. Insoluble materials were removed by filtration. Volatiles were removed under vacuum, and the crude was re-dissolved in CH_2Cl_2 (50 mL) and filtered to remove insoluble materials. The solvent was removed using a rotary evaporator, and the crude was purified by column chromatography on a silica column with gradient mixture of CH_2Cl_2/EtOAc (v/v = 9:1 to 4:6). Fractions containing the product were combined and concentrated using a rotary evaporator to give the product as a pale yellow solid. Yield = 0.73 g, 57%. ^{1}H NMR (400 MHz, CD_3CN, 298 K): δ 7.92–7.64 (m, 4H), 3.68 (t, *J* = 7.0 Hz, 2H), 3.28 (t, *J* = 5.8 Hz, 2H), 2.72 (t, *J* = 5.7 Hz, 2H), 2.61 (t, *J* = 6.7 Hz, 2H), 1.76 (quint, *J* = 6.8 Hz, 2H), 1.34 (s, 1H). ^{13}C{^{1}H} NMR (100 MHz, CD_3CN, 298 K): δ 169.4, 135.0, 133.3, 123.7, 52.1, 49.3, 47.4, 36.8, 29.6. ESI-MS (+ve) calcd. for $C_{13}H_{15}N_5O_2$ $[M+H]^+$ (*m/z*): 273.1, found 273.0.

Synthesis of **2**. A solution of **1** (1.49 g, 5.44 mmol), 37% (w/w) formaldehyde solution (0.88 g, 10.9 mmol), and sodium cyanoborohydride (0.68 g, 10.9 mmol) in MeOH (30 mL) was stirred at room temperature overnight. Deionized water (30 mL) was added and the aqueous layer was extracted with CH_2Cl_2 (50 mL × 2). The organic layers were combined and dried over anhydrous $MgSO_4$. Solvents were removed under vacuum to give the product as a pale yellow solid. Yield = 1.39 g, 89%. ^{1}H NMR (400 MHz, $CDCl_3$, 298 K): δ 7.84 (dd, *J* = 5.5, 3.0 Hz, 2H), 7.71 (dd, *J* = 5.5, 3.0 Hz, 2H), 3.75 (t, *J* = 7.2 Hz, 2H), 3.34 (t, *J* = 6.2 Hz, 2H), 2.61 (t, *J* = 6.2 Hz, 2H), 2.51 (t, *J* = 7.2 Hz, 2H), 2.29 (s, 3H), 1.87 (quint, *J* = 7.2 Hz, 2H). $^{13}C\{^1H\}$ NMR (100 MHz, $CDCl_3$, 298 K): δ 168.5, 134.1, 132.2, 123.4, 56.2, 55.2, 48.4, 41.7, 36.0, 25.8. ESI-MS (+ve) calcd. for $C_{14}H_{17}N_5O_2$ $[M+H]^+$ (*m/z*): 287.1, found 287.0.

Synthesis of **3**. A solution of **2** (0.70 g, 2.44 mmol) and hydrazine hydrate (0.37 g, 7.31 mmol) in MeOH (30 mL) was stirred at room temperature overnight. CH_2Cl_2 (100 mL) was added and insoluble materials were removed by filtration. Volatiles were removed under vacuum, and the crude was re-dissolved in CH_2Cl_2 (50 mL) and filtered to remove insoluble materials. The solvent was removed under vacuum to give the product as a yellow oil. Yield = 0.32 g, 83%. ^{1}H NMR (400 MHz, $CDCl_3$, 298 K): δ 3.34 (t, *J* = 6.1 Hz, 2H), 2.80 (t, *J* = 6.9 Hz, 2H), 2.58 (t, *J* = 6.1 Hz, 2H), 2.47 (t, *J* = 7.0 Hz, 2H), 2.27 (s, 3H), 2.08 (s, 2H), 1.65 (quint, *J* = 6.9 Hz, 2H). $^{13}C\{^1H\}$ NMR (100 MHz, $CDCl_3$, 298 K): δ 56.7, 55.8, 49.1, 42.2, 40.5, 31.0. ESI-MS (+ve) calcd. for $C_6H_{15}N_5$ $[M+H]^+$ (*m/z*): 157.1, found 157.0.

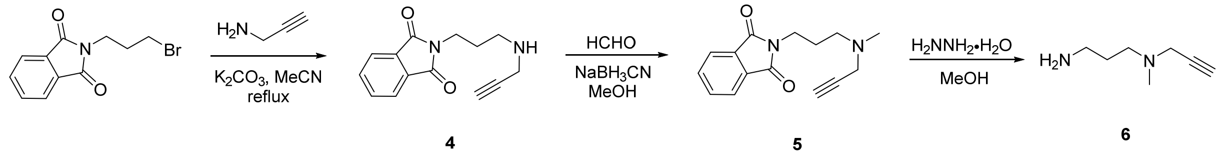

4 **5** **6**

Synthesis of **4**. A mixture of *N*-(3-bromopropyl)phthalimide (2.00 g, 7.49 mmol), propargylamine (0.82 g, 15.0 mmol), and potassium carbonate (3.10 g, 22.5 mmol) was refluxed in MeCN (150 mL) overnight. Insoluble materials were removed by filtration. Volatiles were removed under vacuum, and the crude was re-dissolved in CH_2Cl_2 (50 mL) and filtered to remove insoluble materials. The solvent was removed using a rotary evaporator, and the crude was purified by column chromatography on a silica column with gradient mixture of CH_2Cl_2/EtOAc (v/v = 8:2 to 3:7). Fractions containing the product were combined and concentrated using a rotary evaporator to afford the product as a pale yellow solid. Yield = 0.94 g, 52%. ^{1}H NMR (400 MHz, CD_3CN, 298 K): δ 7.92–7.58 (m, 4H), 3.67 (t, *J* = 7.0 Hz, 2H), 3.33 (d, *J* = 2.4 Hz, 2H), 2.64 (t, *J* = 6.8 Hz, 2H), 2.37 (t, *J* = 2.4 Hz, 1H), 1.76 (quint, *J* = 6.9 Hz, 2H), 1.37 (s, 1H). $^{13}C\{^1H\}$ NMR (100 MHz, CD_3CN, 298 K): δ 169.4, 135.0, 133.3, 123.7, 83.6, 72.1, 46.5, 38.4, 36.8, 29.2. ESI-MS (+ve) calcd. for $C_{14}H_{14}N_2O_2$ $[M+H]^+$ (*m/z*): 242.1, found 242.0.

Synthesis of **5**. A solution of **4** (0.94 g, 3.88 mmol), 37% (w/w) formaldehyde solution (0.94 g, 11.6 mmol), and sodium cyanoborohydride (0.73 g, 11.6 mmol) in MeOH (30 mL) was stirred at room temperature overnight. Deionized water (30 mL) was added and the aqueous layer was extracted with CH_2Cl_2 (50 mL × 2). The organic layers were combined and dried over anhydrous $MgSO_4$. Solvents were removed under vacuum to afford the product as a pale yellow solid. Yield = 0.91 g, 92%. ^{1}H NMR (400 MHz, $CDCl_3$, 298 K): δ 7.88–7.79 (m, 2H), 7.76–7.66 (m, 2H), 3.75 (t, *J* = 7.2 Hz, 2H), 3.37 (s, 2H), 2.53 (t, *J* = 7.1, 2H), 2.31 (s, 3H), 2.18 (s, 1H), 1.86 (quint, *J* = 7.2 Hz, 2H). ^{13}C{^{1}H} NMR (100 MHz, $CDCl_3$, 298 K): δ 168.5, 134.0, 132.3, 123.3, 73.6, 52.9, 45.5, 43.6, 41.6, 36.3, 26.4. ESI-MS (+ve) calcd. for $C_{15}H_{16}N_2O_2$ $[M+H]^+$ (*m/z*): 256.1, found 256.0.

Synthesis of **6**. Under an argon atmosphere, a solution of **5** (0.91 g, 3.56 mmol) and hydrazine hydrate (0.53 g, 10.7 mmol) in MeOH (30 mL) was stirred at room temperature overnight. CH_2Cl_2 (100 mL) was added and insoluble materials were removed by filtration. Volatiles were removed under vacuum, and the crude was re-dissolved in CH_2Cl_2 (50 mL) and filtered to remove insoluble materials. The solvent was removed under vacuum to afford the product as a yellow oil. Yield = 0.29 g, 64%. ^{1}H NMR (400 MHz, $CDCl_3$, 298 K): δ 3.34 (d, *J* = 2.4 Hz, 2H), 2.77 (t, *J* = 6.8 Hz, 2H), 2.49 (t, *J* = 7.2 Hz, 2H), 2.30 (s, 3H), 2.21 (t, *J* = 2.4 Hz, 1H), 2.05 (s, 2H), 1.62 (quint, *J* = 6.9 Hz, 2H). ^{13}C{^{1}H} NMR (100 MHz, $CDCl_3$, 298 K): δ 78.7, 73.2, 53.6, 45.8, 41.9, 40.6, 30.8. ESI-MS (+ve) calcd. for $C_7H_{14}N_2$ $[M+H]^+$ (*m/z*): 126.1, found 126.0.

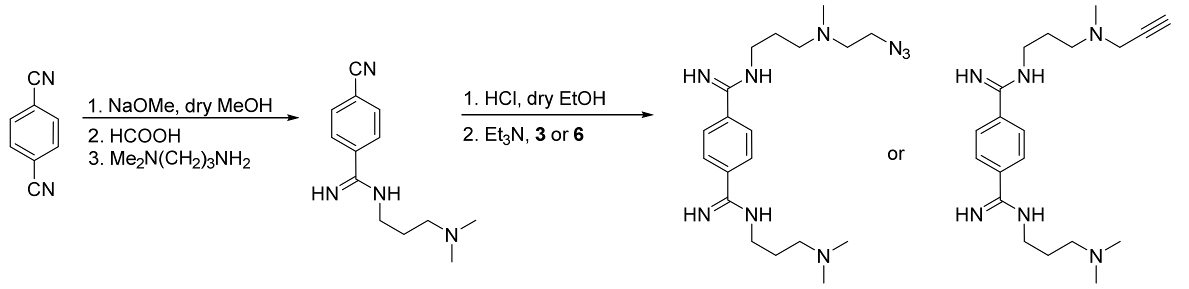

DB-CN, 56% **DB-N$_3$**, 41% **DB-alkyne**, 34%

DB-CN. To a suspension of sodium methoxide (0.84 g, 15.6 mmol) in dry methanol (30 mL), 1,4-dicyanobenzene (1.00 g, 7.80 mmol) was added and the mixture was stirred at room temperature overnight. Formic acid (1.08 g, 23.4 mmol) was added to give a colourless solution, followed by the addition of *N*,*N*-dimethyl-1,3-diaminopropane (0.80 g, 7.80 mmol), and the resulting solution was stirred at room temperature overnight. Volatiles were removed under vacuum and the solid was re-dissolved in CH_2Cl_2 (50 mL). Insoluble materials were removed by filtration and the solvent was removed using a rotary evaporator. The crude was purified by column chromatography on a neutral aluminium oxide column with gradient mixture of CH_2Cl_2/MeOH (v/v = 99:1 to 95:5).

Fractions containing the product were combined and concentrated using a rotary evaporator to afford the product as a pale yellow solid. Yield = 1.01 g, 56%. ^{1}H NMR (400 MHz, DMSO-d_6, 298 K): δ 8.00 (d, *J* = 8.6 Hz, 2H), 7.93 (d, *J* = 8.5 Hz, 2H), 3.29 (t, *J* = 6.8 Hz, 2H), 2.32 (t, *J* = 6.9 Hz, 2H), 2.16 (s, 6H), 1.75 (quint, *J* = 6.9 Hz, 2H). ^{13}C{^{1}H} NMR (125 MHz, DMSO-d_6, 298 K): δ 159.5, 132.4, 128.6, 128.3, 118.2, 113.9, 56.4, 48.6, 45.0, 26.3. ESI-MS (+ve) calcd. for $C_{13}H_{18}N_4$ $[M+H]^+$ (*m/z*): 231.2, found 231.1.

DB-N$_3$. A solution of **DB-CN** (0.24 g, 1.02 mmol) in 10 mL of dry, HCl-saturated ethanol was stirred at room temperature overnight. Dry diethyl ether (20 mL) was added and the mixture was centrifuged (4000 rpm, 3 min). The solution was decanted and the residue was washed with dry diethyl ether (10 mL × 3) and re-dissolved in dry EtOH (25 mL). To the solution, triethylamine (0.41 g, 2.09 mmol) was added, followed by the addition of **3** (0.19 g, 1.23 mmol), and the resulting solution was stirred at room temperature for 18 hours. Volatiles were removed under vacuum and the crude was purified by column chromatography on a basic aluminium oxide column with gradient mixture of CH_2Cl_2/MeOH (v/v = 95:5 to 90:10). Fractions containing the product were combined and concentrated using a rotary evaporator to give the product as a pale yellow solid. Yield = 0.16 g, 41%. ^{1}H NMR (500 MHz, CD_3OD, 298 K): δ 7.68 (s, 4H), 3.39 (t, *J* = 6.1 Hz, 2H), 3.33–3.24 (m, 4H), 2.60 (t, *J* = 6.1 Hz, 2H), 2.54 (t, *J* = 7.2 Hz, 2H), 2.47–2.40 (m, 2H), 2.30 (s, 3H), 2.26 (s, 6H), 1.85 (quint, *J* = 7.0 Hz, 4H). ^{13}C{^{1}H} NMR (100 MHz, CD_3OD, 298 K): δ 163.9, 163.8, 140.1, 140.0, 128.0, 58.5, 57.2, 56.8, 49.9, 45.5, 42.9, 42.8, 42.4, 28.0, 27.5. HRMS (ESI+) calcd. for $C_{19}H_{33}N_9$ $[M+H]^+$ (*m/z*): 388.2932, found: 388.2930.

DB-alkyne. A solution of **DB-CN** (0.34 g, 1.48 mmol) in 10 mL of dry, HCl-saturated ethanol was stirred at room temperature overnight. Dry diethyl ether (20 mL) was added and the mixture was centrifuged (4000 rpm, 3 min). The solution was decanted and the residue was washed with dry diethyl ether (10 mL × 3) and re-dissolved in dry EtOH (25 mL). To the solution, triethylamine (0.60 g, 5.93 mmol) was added, followed by the addition of **6** (0.23 g, 1.83 mmol), and the resulting solution was stirred at room temperature for 18 hours. Volatiles were removed under vacuum and the crude was purified by column chromatography on a basic aluminium oxide column with gradient mixture of CH_2Cl_2/MeOH (v/v = 95:5 to 90:10). Fractions containing the product were combined and concentrated using a rotary evaporator to give the product as a pale yellow solid. Yield = 0.18 g, 34%. ^{1}H NMR (400 MHz, CD_3OD, 298 K): δ 7.67 (s, 4H), 3.35 (s, 2H), 3.30 – 3.21 (m, 4H), 2.67 (t, *J* = 2.4 Hz, 1H), 2.56 (t, *J* = 7.3 Hz, 2H), 2.41 (t, *J* = 7.6 Hz, 2H), 2.32 (s, 3H), 2.24 (s, 6H), 1.89 – 1.76 (m, 4H). ^{13}C{^{1}H} NMR (100 MHz, CD_3OD, 298 K): δ 163.5, 163.4, 140.0, 140.0, 127.9, 79.1, 75.3, 58.5, 54.7, 46.1, 45.6, 43.0, 42.8, 42.0, 28.0, 27.8. HRMS (ESI+) calcd. for $C_{20}H_{32}N_6$ $[M+H]^+$ (*m/z*): 357.2761, found: 357.2763.

Synthesis of **Res-alkyne**. To a suspension of resorufin sodium salt (0.50 g, 2.14 mmol) in DMF (20 mL), potassium carbonate (0.44 g, 3.22 mmol) and potassium iodide (36 mg, 0.21 mmol) were added, followed by the addition of 80% (w/w) propargyl bromide in toluene (0.64 g, 4.29 mmol), and the resulting mixture was heated at 70°C overnight. The mixture was cooled to room temperature, and a 20% (w/v) potassium carbonate solution (100 mL) was added. The orange-red solid was collected by vacuum filtration, washed with deionized water (50 mL × 3), and dried under vacuum. Yield = 0.27 g, 49%. ^{1}H NMR (500 MHz, DMSO-d_6, 298 K): δ 7.81 (d, J = 8.8 Hz, 1H), 7.54 (d, J = 9.8 Hz, 1H), 7.17 (d, J = 2.7 Hz, 1H), 7.10 (dd, J = 8.9, 2.6 Hz, 1H), 6.80 (dd, J = 9.9, 1.8 Hz, 1H), 6.29 (d, J = 1.9 Hz, 1H), 5.00 (d, J = 2.0 Hz, 2H), 3.70 (t, J = 2.1 Hz, 1H). $^{13}C\{^{1}H\}$ NMR (125 MHz, DMSO-d_6, 298 K): δ 185.5, 161.0, 149.7, 145.7, 145.1, 135.0, 133.9, 131.3, 128.3, 114.2, 105.8, 101.4, 79.2, 78.3, 56.6. HR-ESI-MS (+ve) calcd. for $C_{15}H_{10}NO_3$ $[M+H]^+$ (m/z): 252.0655, found: 252.0652.

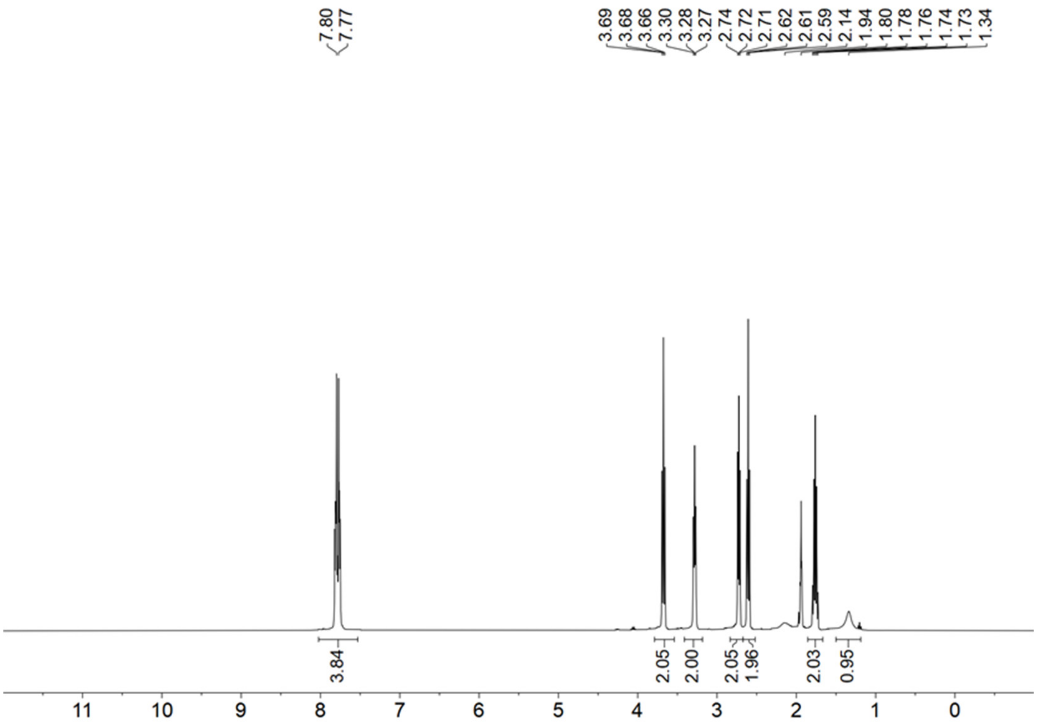

Figure S1. ^{1}H NMR (400 MHz, CD_3CN, 298 K) spectrum of **1**.

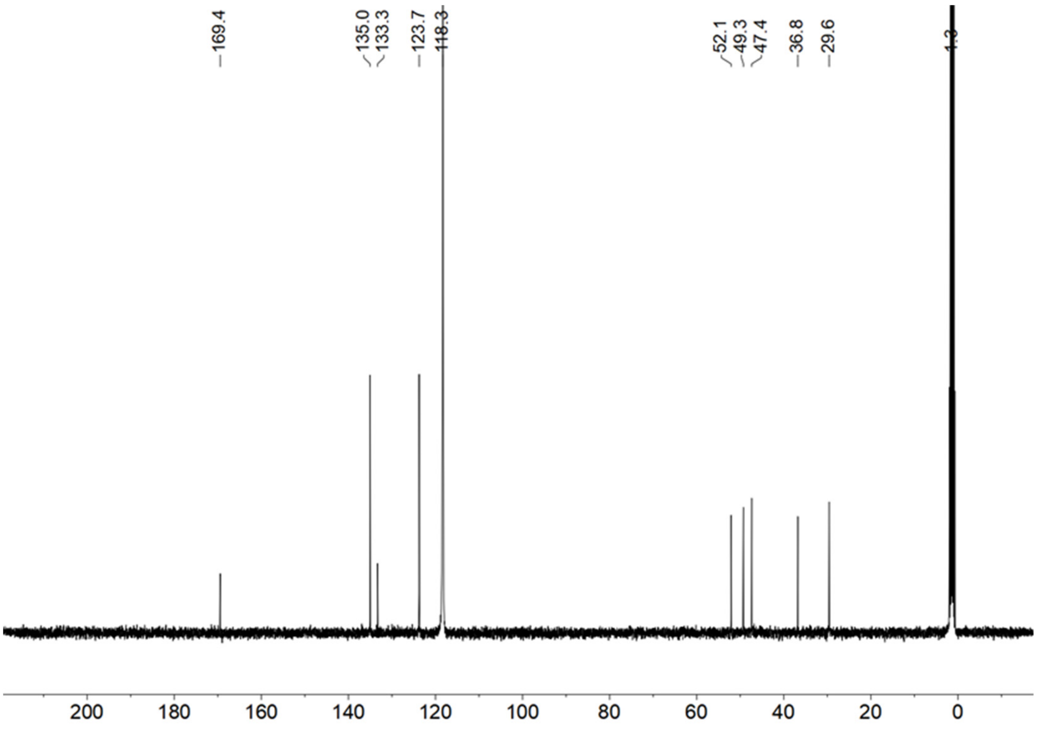

Figure S2. $^{13}C\{^{1}H\}$ NMR (100 MHz, CD_3CN, 298 K) spectrum of **1**.

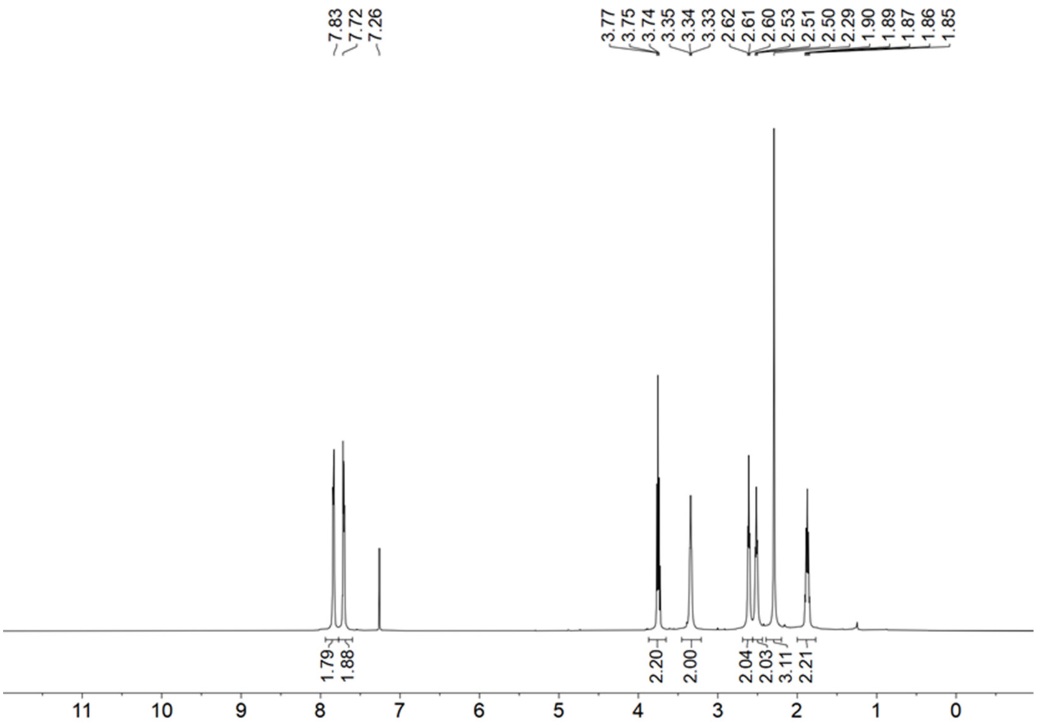

Figure S3. ^{1}H NMR (400 MHz, $CDCl_3$, 298 K) spectrum of **2**.

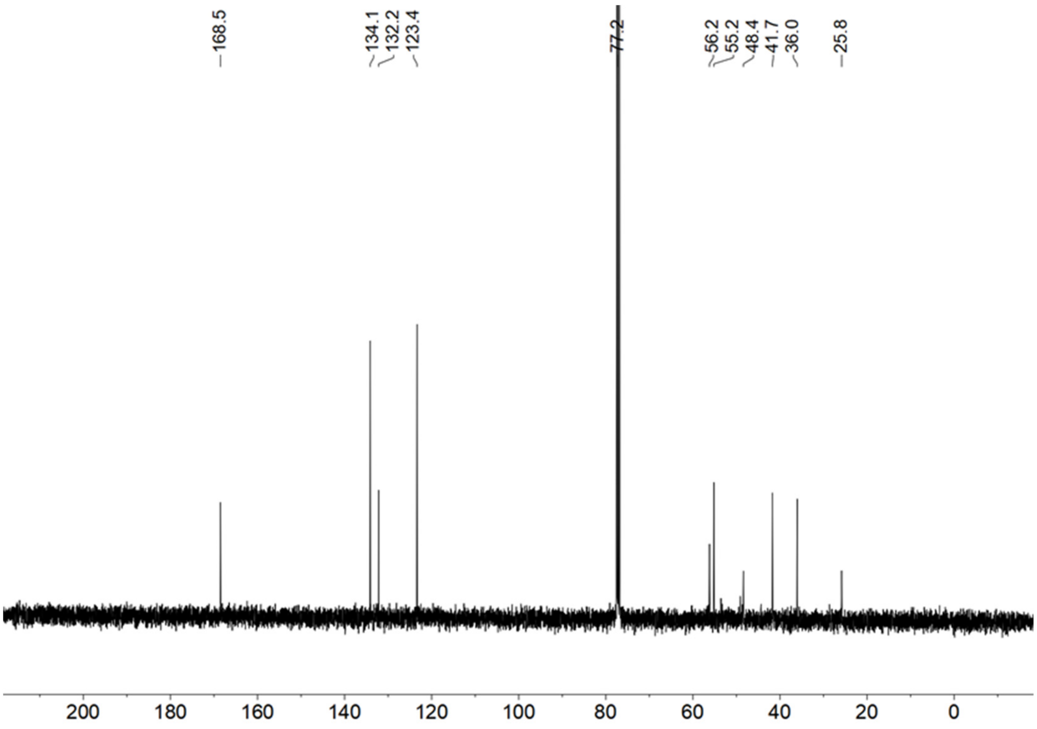

Figure S4. $^{13}C\{^{1}H\}$ NMR (100 MHz, $CDCl_3$, 298 K) spectrum of **2**.

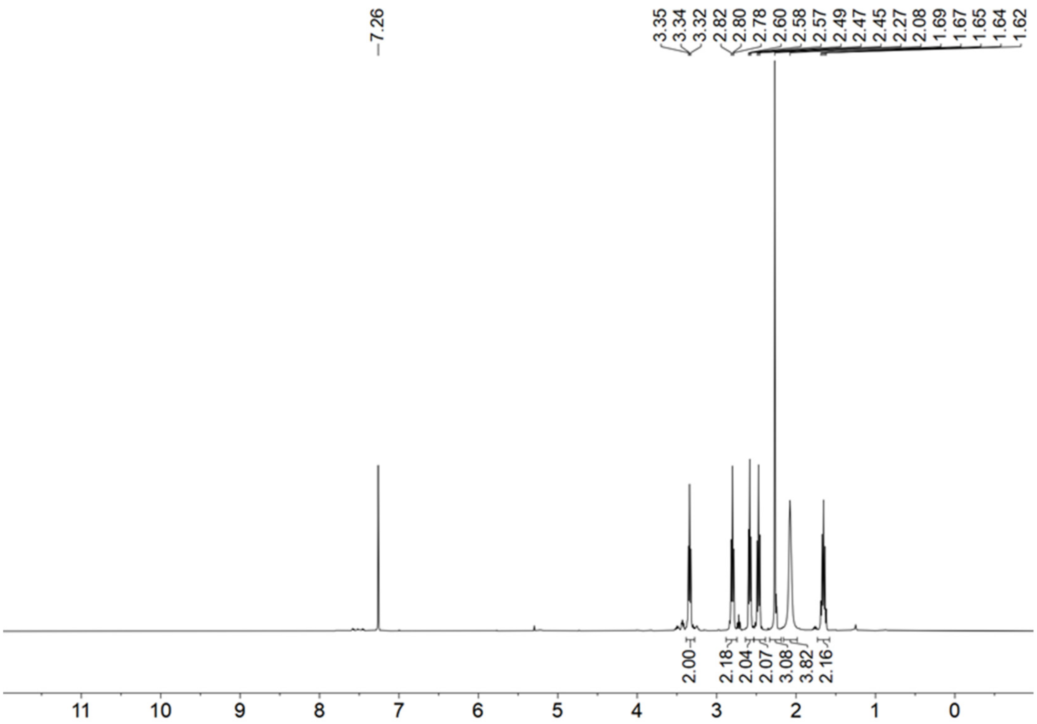

Figure S5. ^{1}H NMR (400 MHz, $CDCl_3$, 298 K) spectrum of **3**.

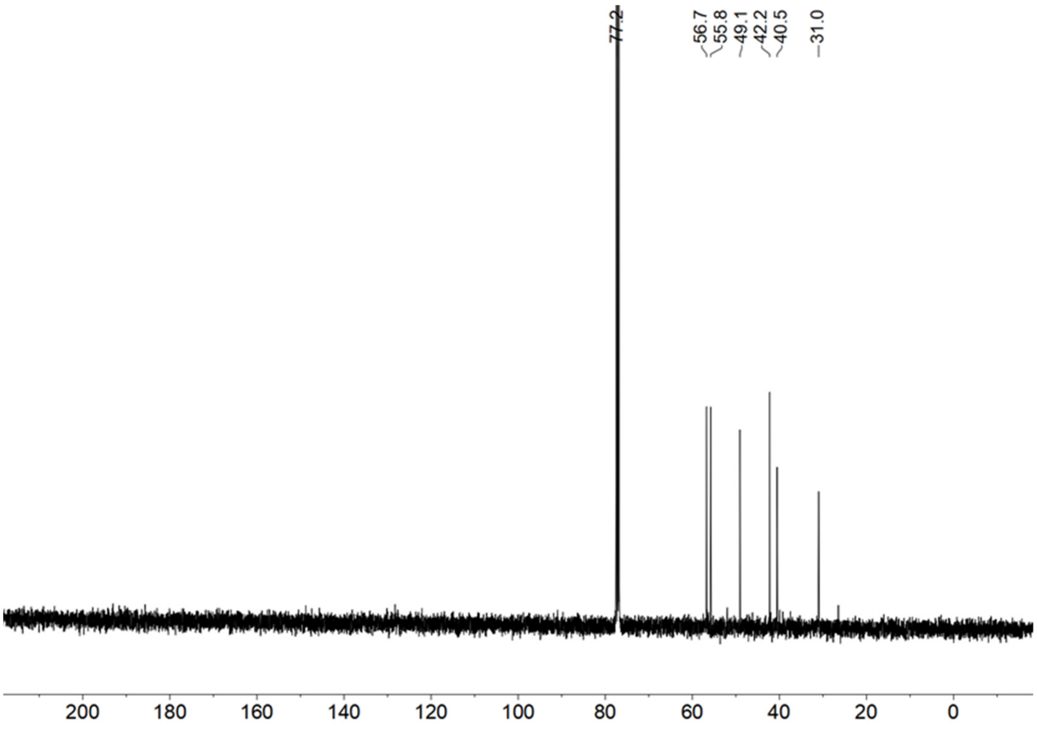

Figure S6. $^{13}C\{^{1}H\}$ NMR (100 MHz, $CDCl_3$, 298 K) spectrum of **3**.

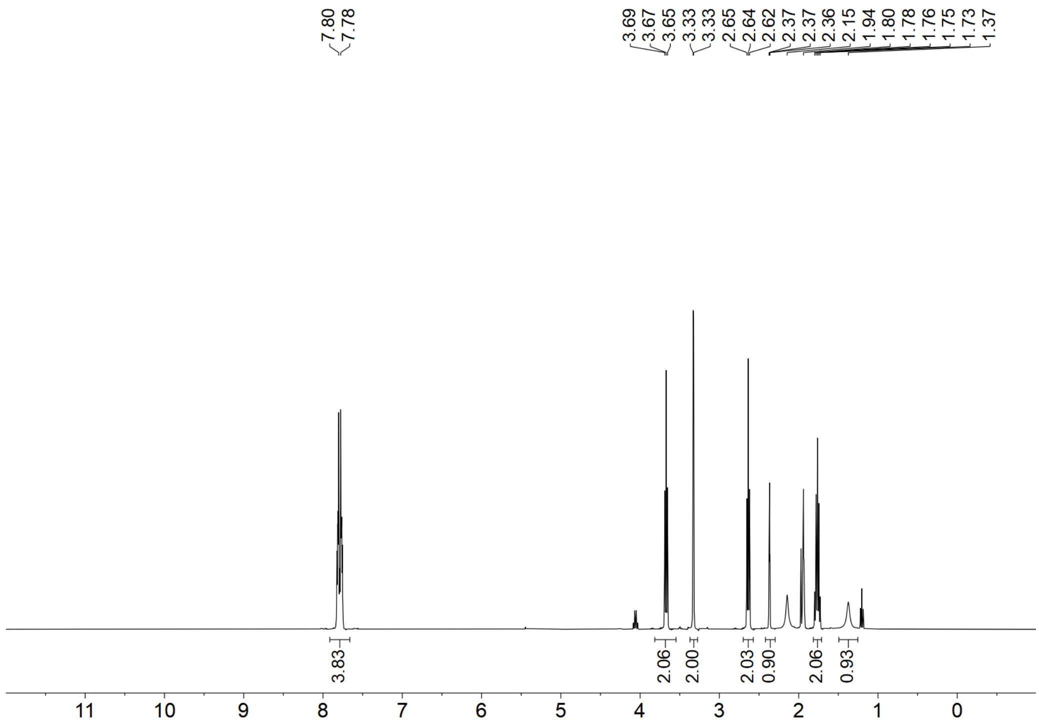

Figure S7. ¹H NMR (400 MHz, CD_3CN, 298 K) spectrum of **4**.

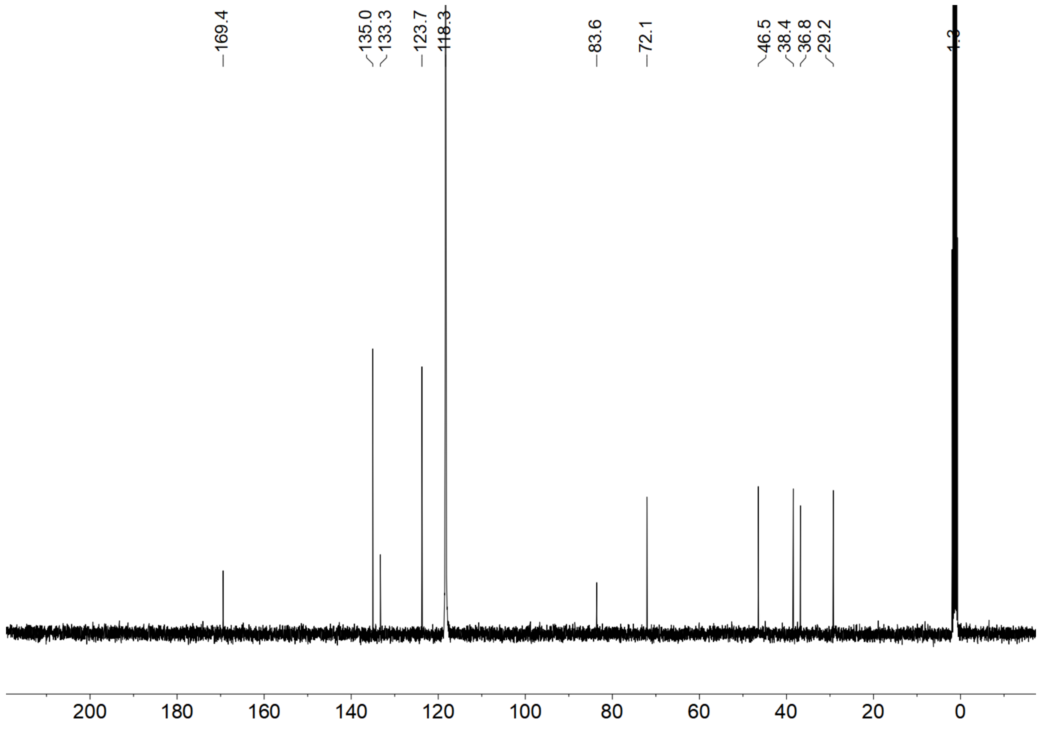

Figure S8. ¹³C{¹H} NMR (100 MHz, CD_3CN, 298 K) spectrum of **4**.

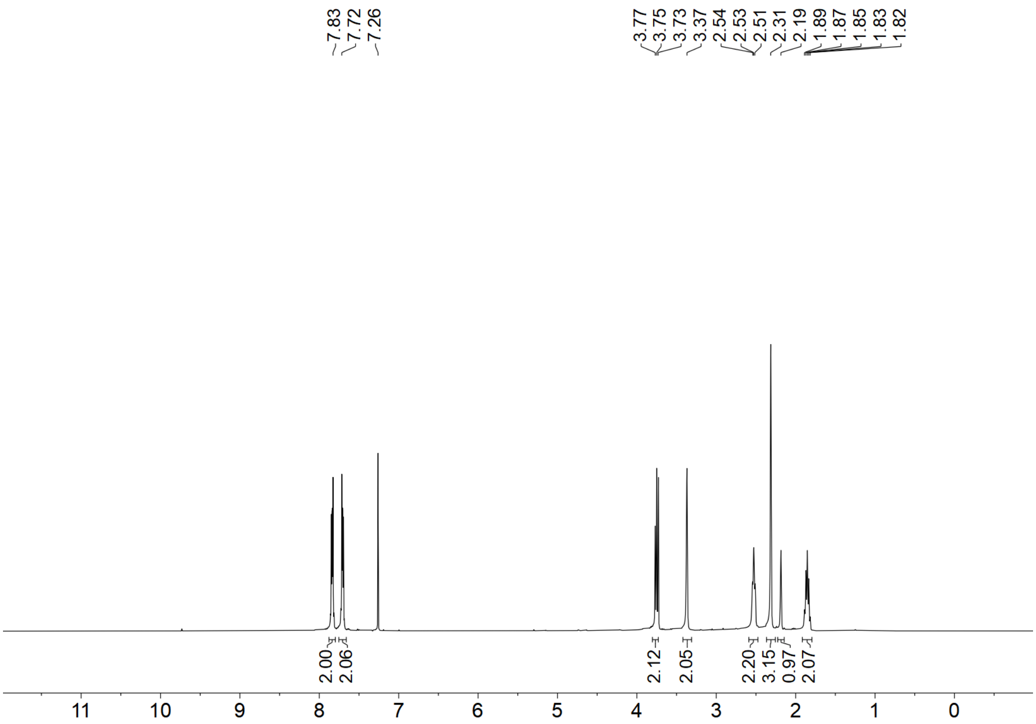

Figure S9. ^{1}H NMR (400 MHz, $CDCl_3$, 298 K) spectrum of **5**.

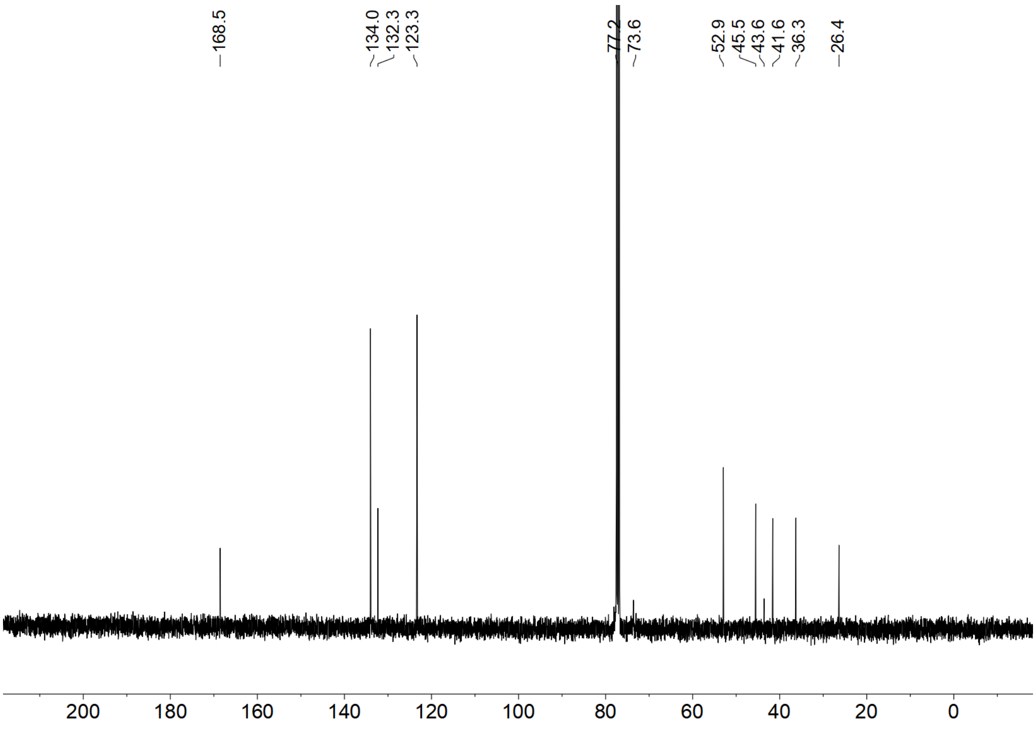

Figure S10. $^{13}C\{^{1}H\}$ NMR (100 MHz, $CDCl_3$, 298 K) spectrum of **5**.

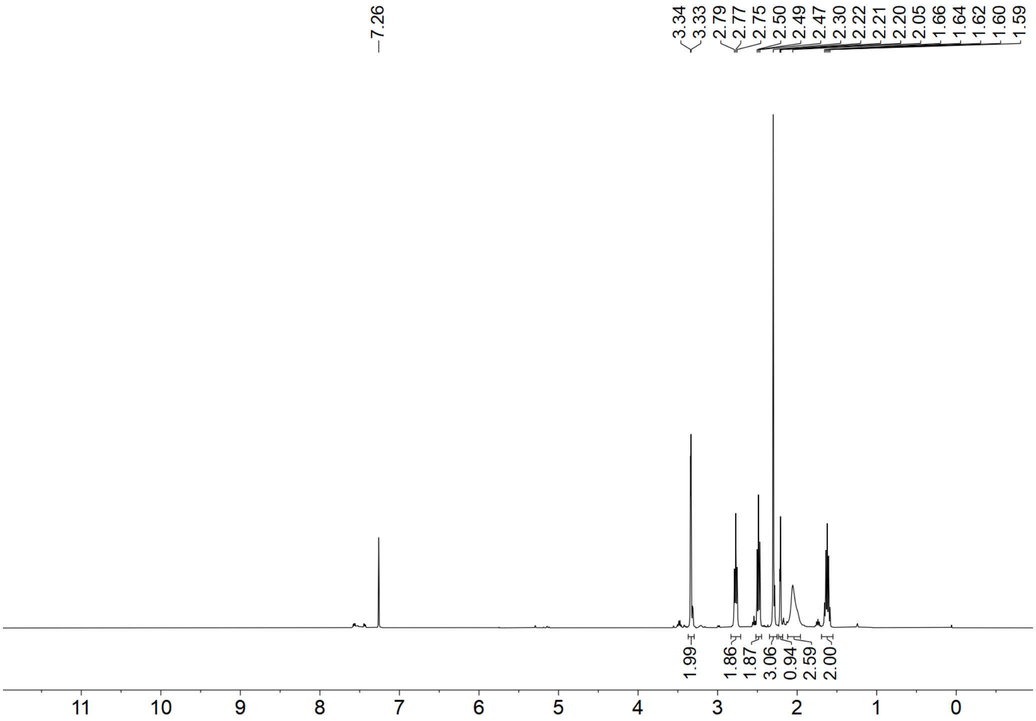

Figure S11. ^{1}H NMR (400 MHz, $CDCl_3$, 298 K) spectrum of **6**.

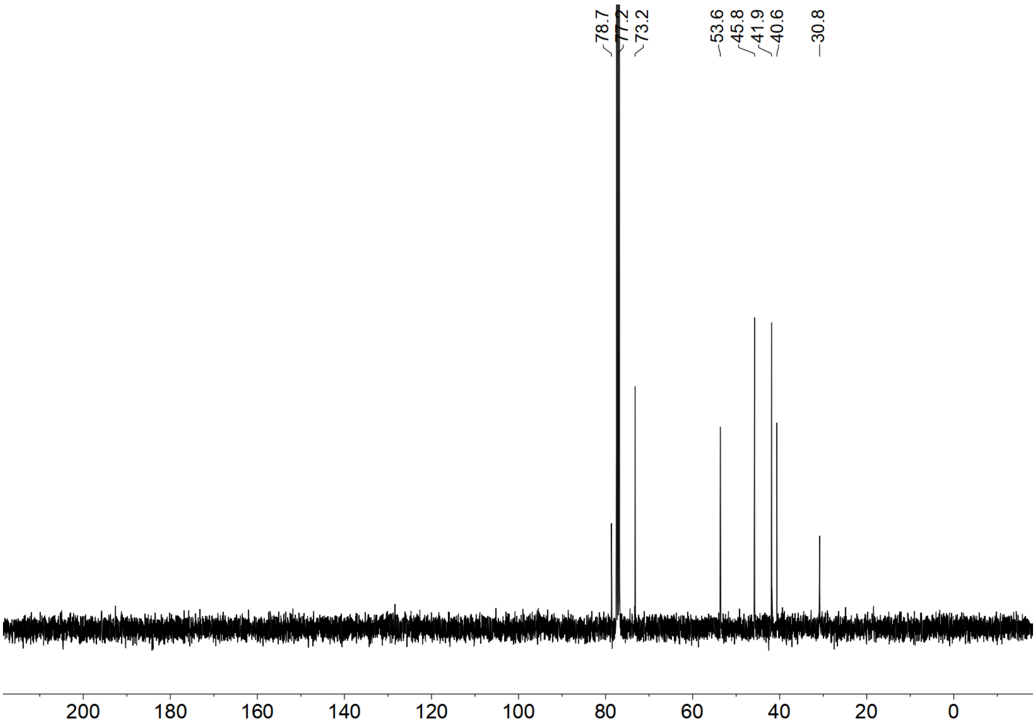

Figure S12. $^{13}C\{^{1}H\}$ NMR (100 MHz, $CDCl_3$, 298 K) spectrum of **6**.

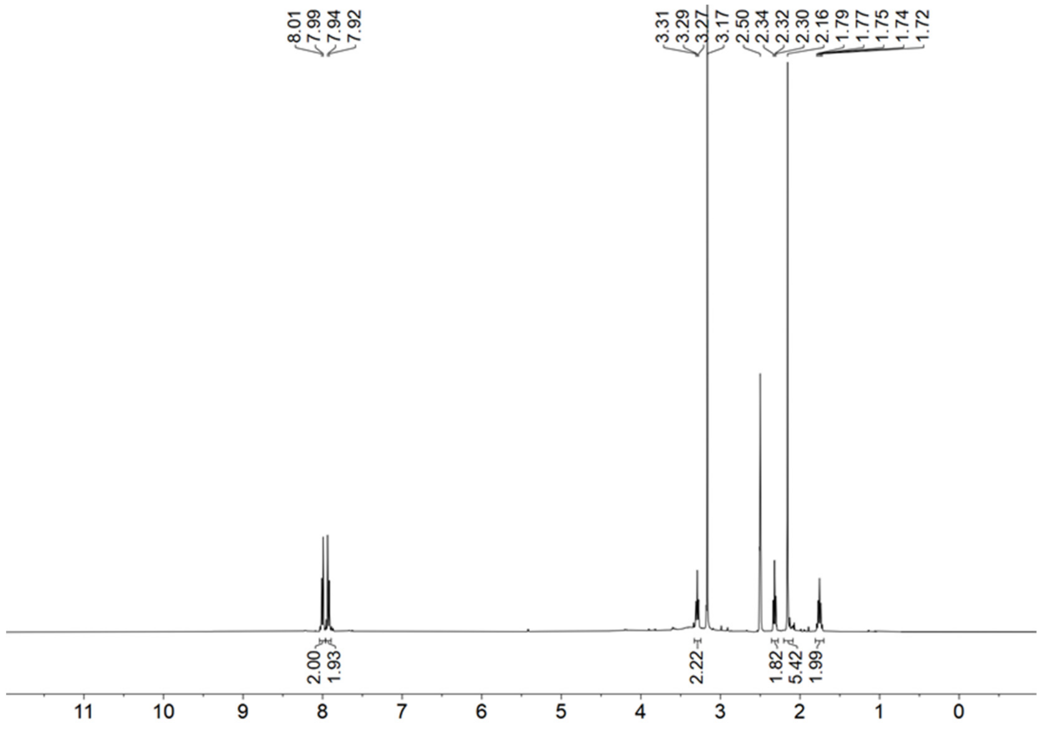

Figure S13. ^{1}H NMR (400 MHz, DMSO-d_6, 298 K) spectrum of **DB-CN**.

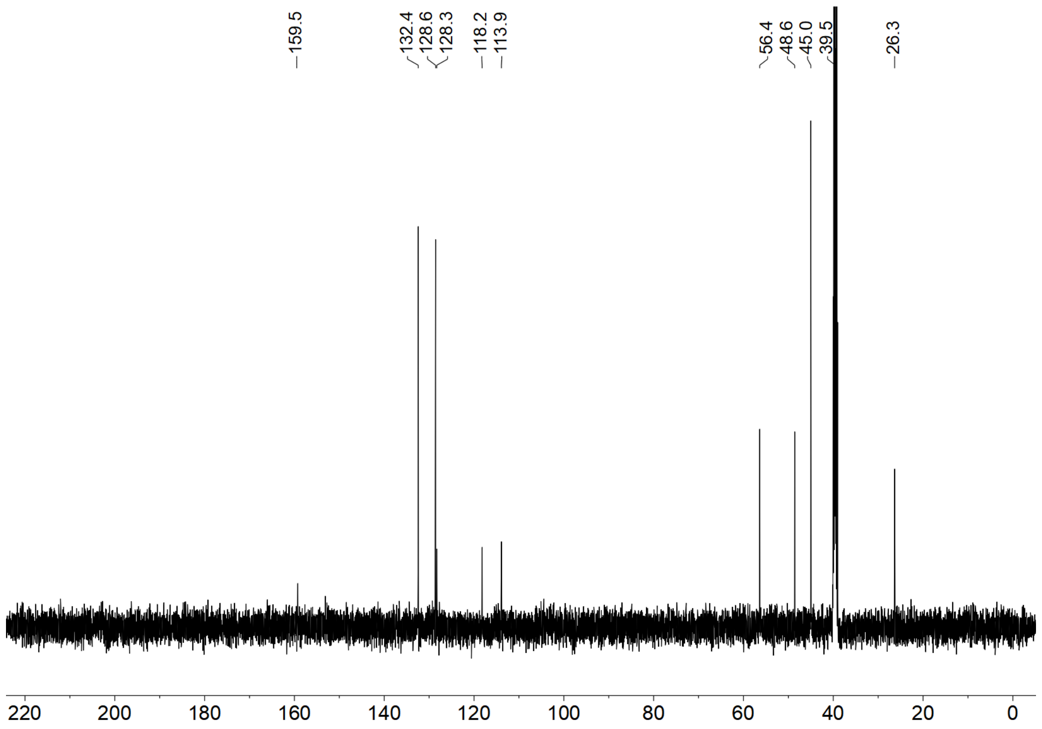

Figure S14. $^{13}C\{^{1}H\}$ NMR (125 MHz, DMSO-d_6, 298 K) spectrum of **DB-CN**.

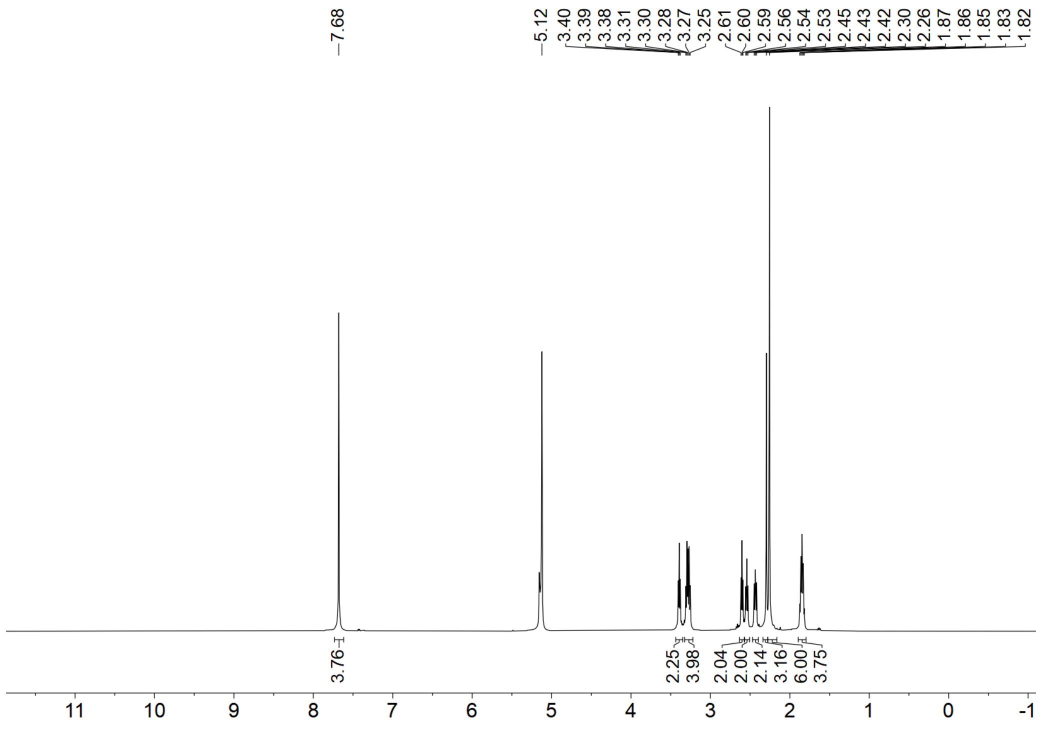

Figure S15. ^1H NMR (400 MHz, CD_3OD, 298 K) spectrum of **DB-N$_3$**.

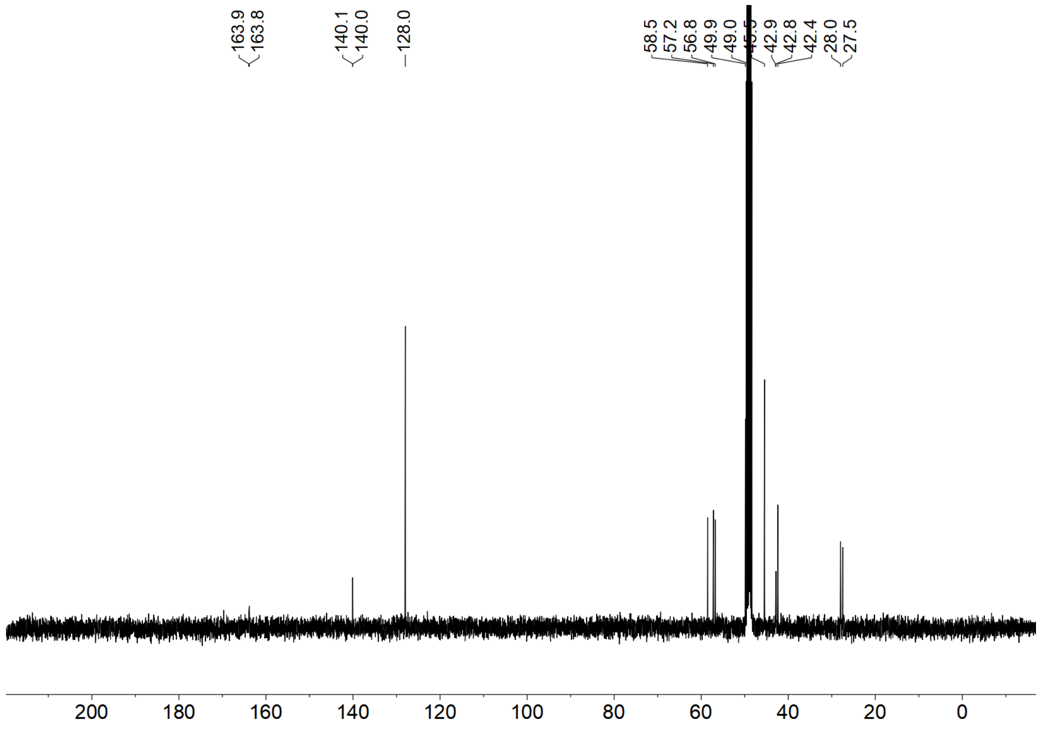

Figure S16. $^{13}C\{^1H\}$ NMR (100 MHz, CD_3OD, 298 K) spectrum of **DB-N$_3$**.

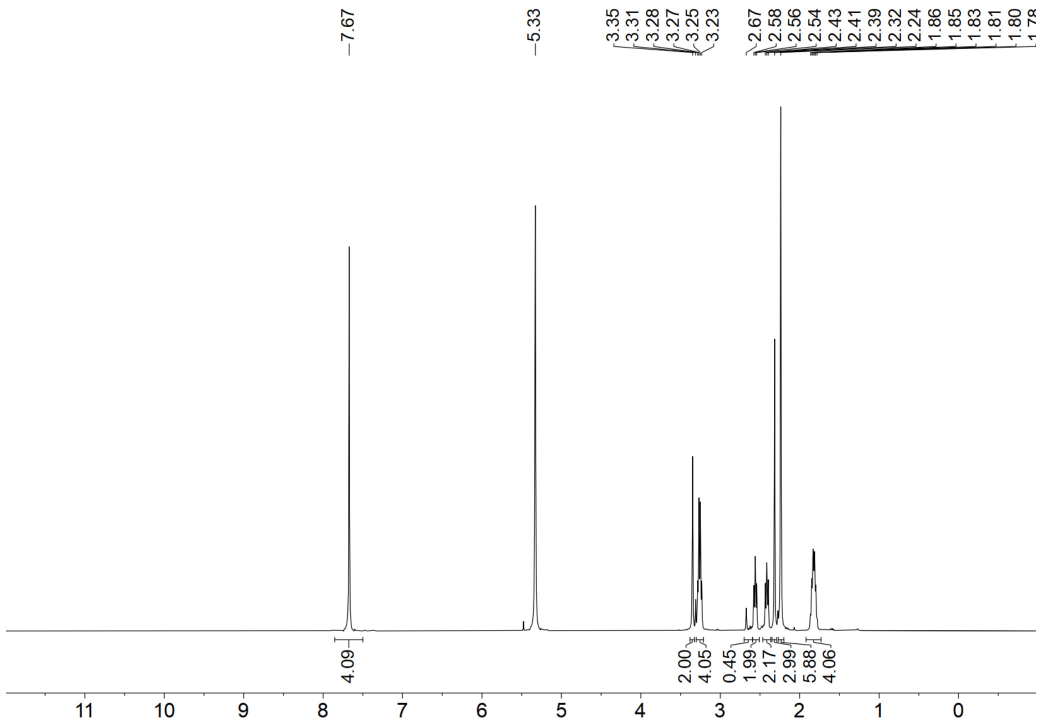

Figure S17. ^{1}H NMR (400 MHz, CD_3OD, 298 K) spectrum of **DB-alkyne**.

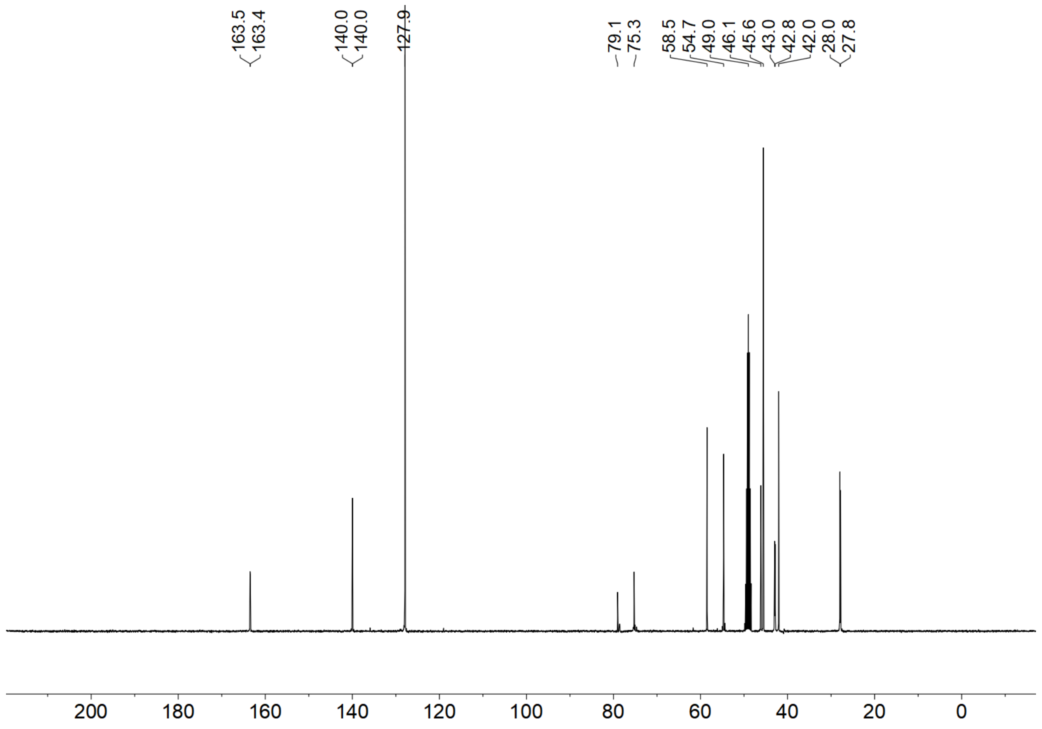

Figure S18. $^{13}C\{^{1}H\}$ NMR (100 MHz, CD_3OD, 298 K) spectrum of **DB-alkyne**.

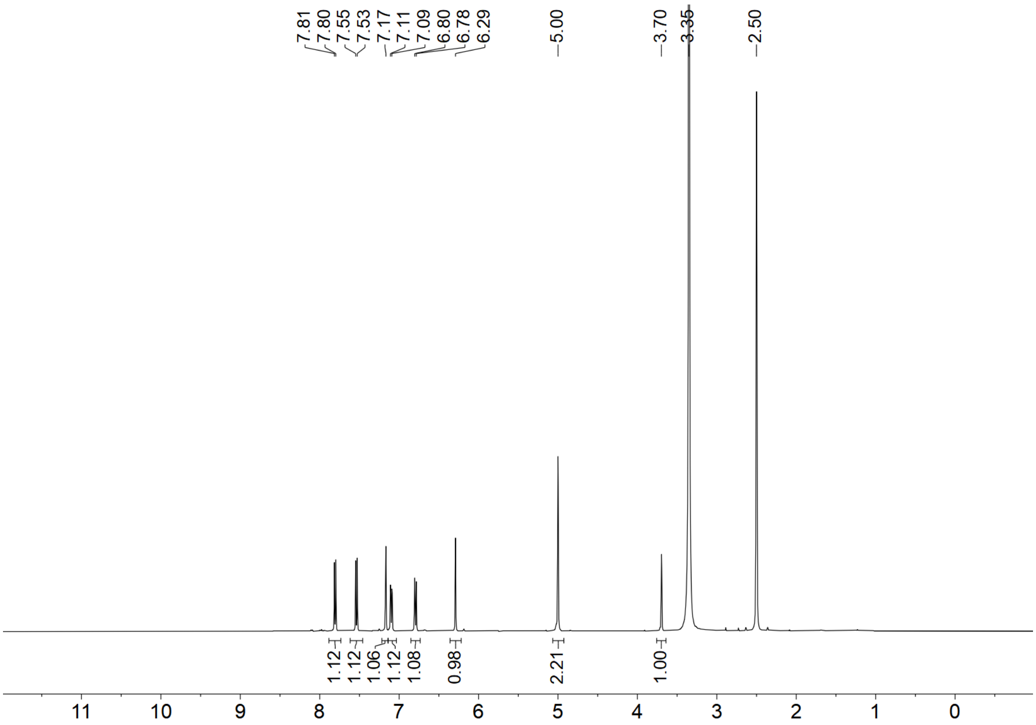

Figure S19. ^{1}H NMR (500 MHz, DMSO-d_6, 298 K) spectrum of **Res-alkyne**.

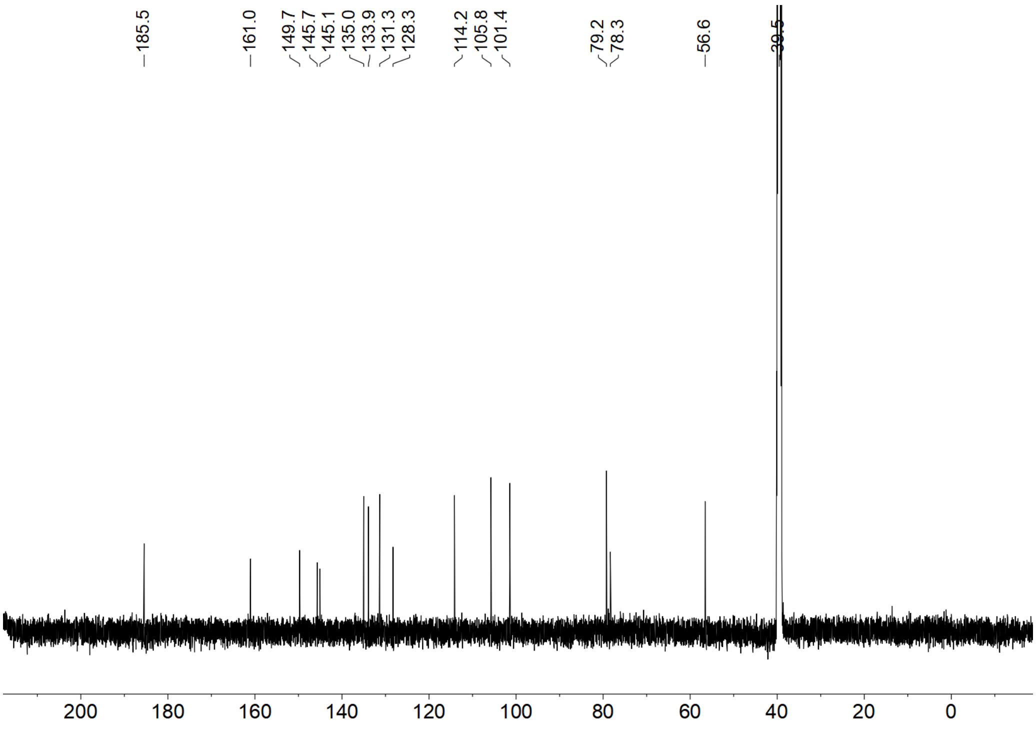

Figure S20. ^{13}C{^{1}H} NMR (125 MHz, DMSO-d_6, 298 K) spectrum of **Res-alkyne**.

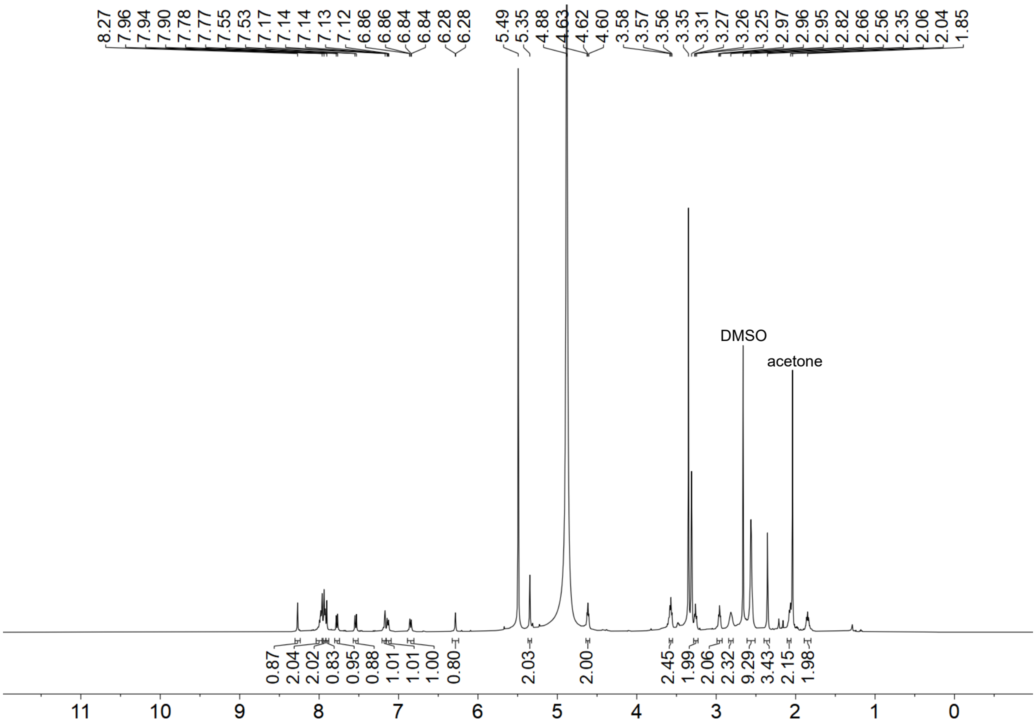

Figure S21. ^{1}H NMR (500 MHz, CD_3OD, 298 K) spectrum of **DB-Res**.

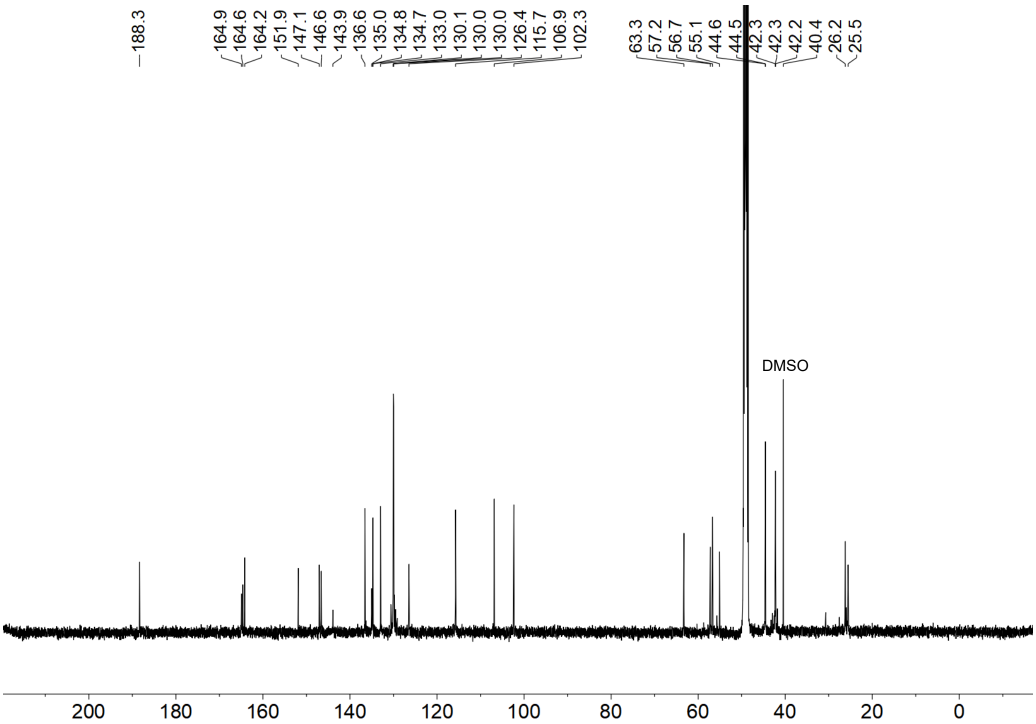

Figure S22. $^{13}C\{^1H\}$ NMR (125 MHz, CD_3OD, 298 K) spectrum of **DB-Res**.

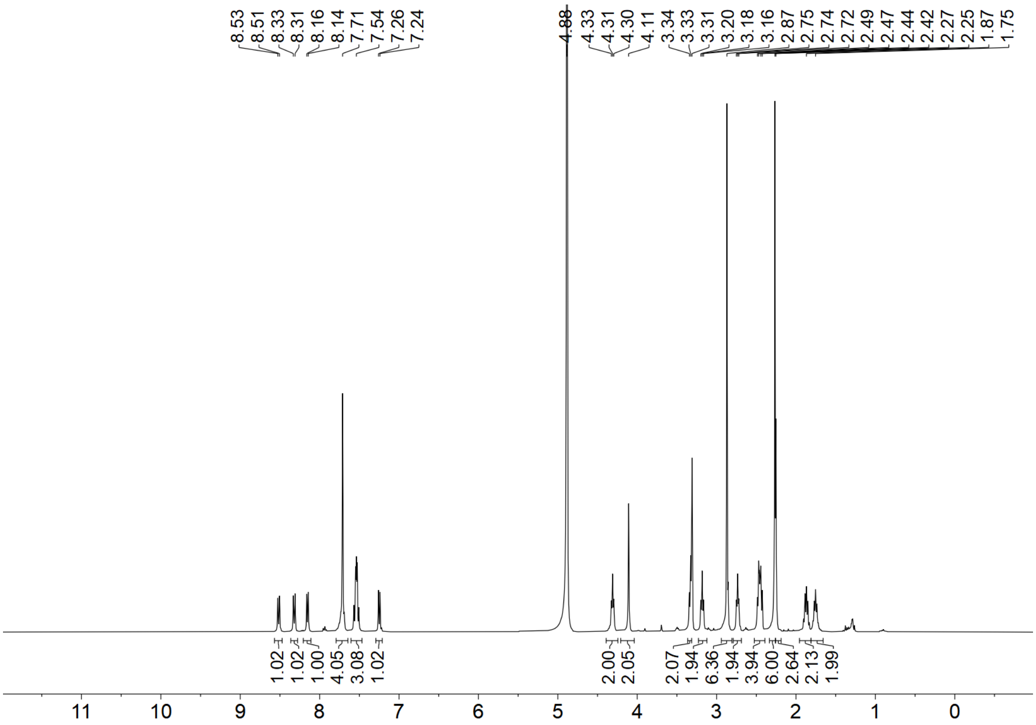

Figure S23. ^{1}H NMR (400 MHz, CD_3OD, 298 K) spectrum of **DB-Dan**.

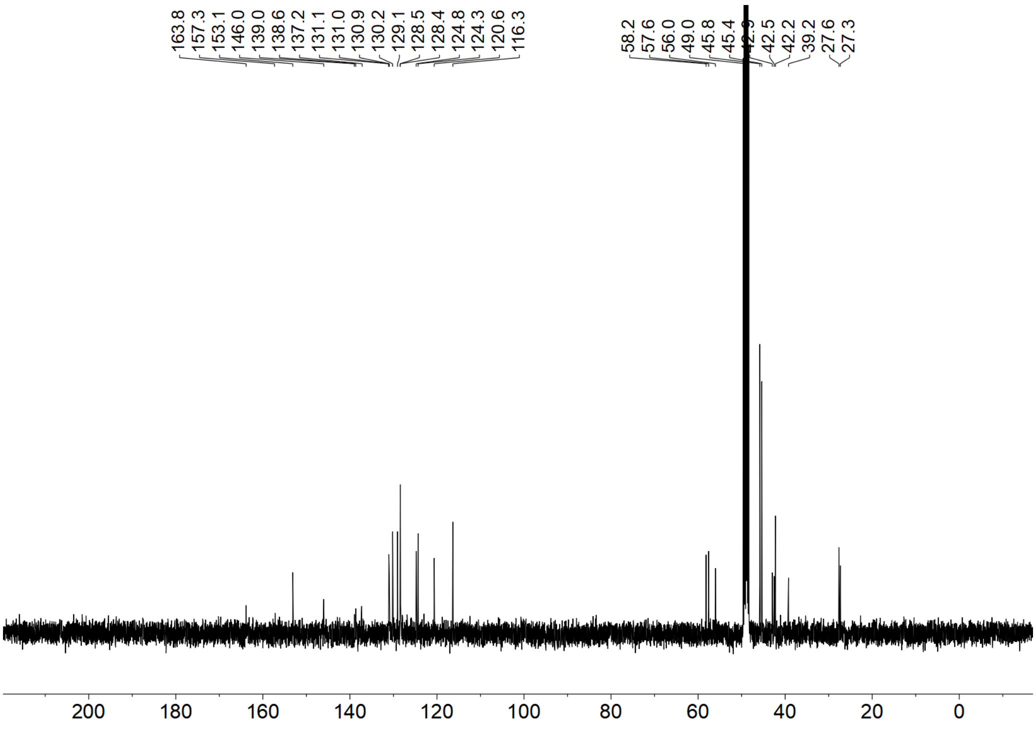

Figure S24. ^{13}C{^{1}H} NMR (125 MHz, CD_3OD, 298 K) spectrum of **DB-Dan**.

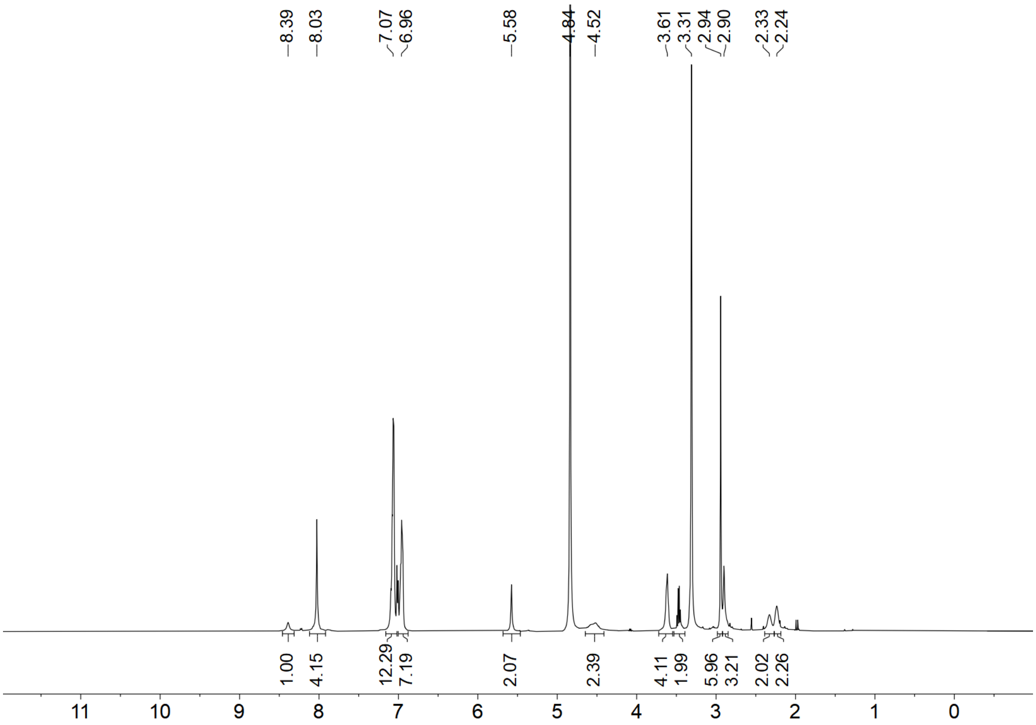

Figure S25. ^{1}H NMR (400 MHz, CD_3OD, 298 K) spectrum of **DB-TPE**.

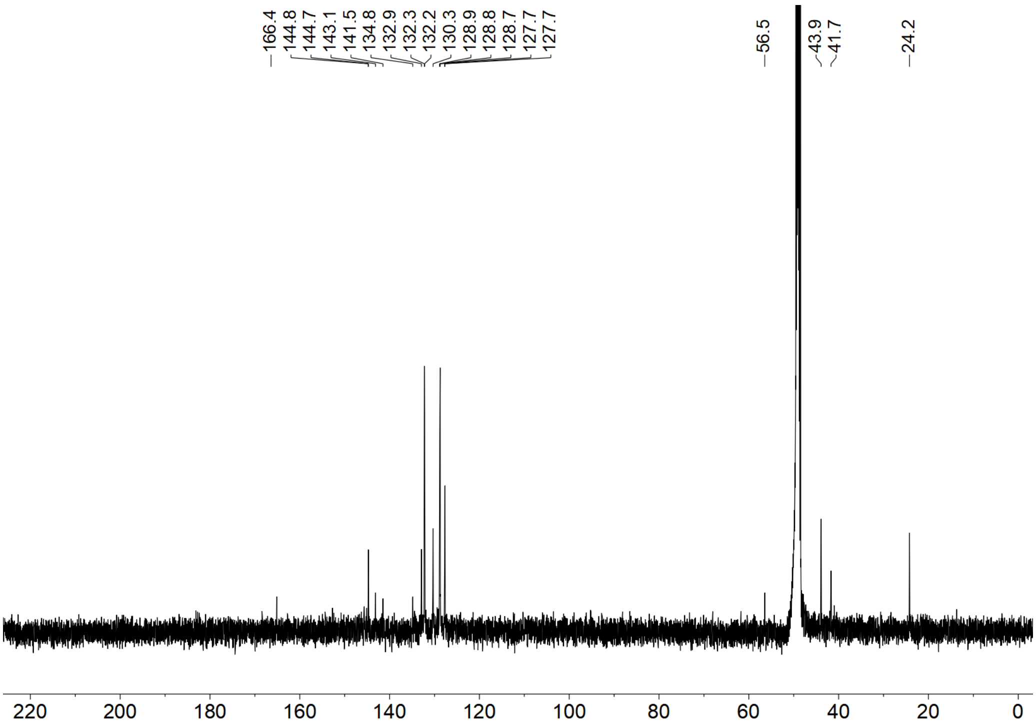

Figure S26. $^{13}C\{^{1}H\}$ NMR (125 MHz, CD_3OD, 298 K) spectrum of **DB-TPE**.

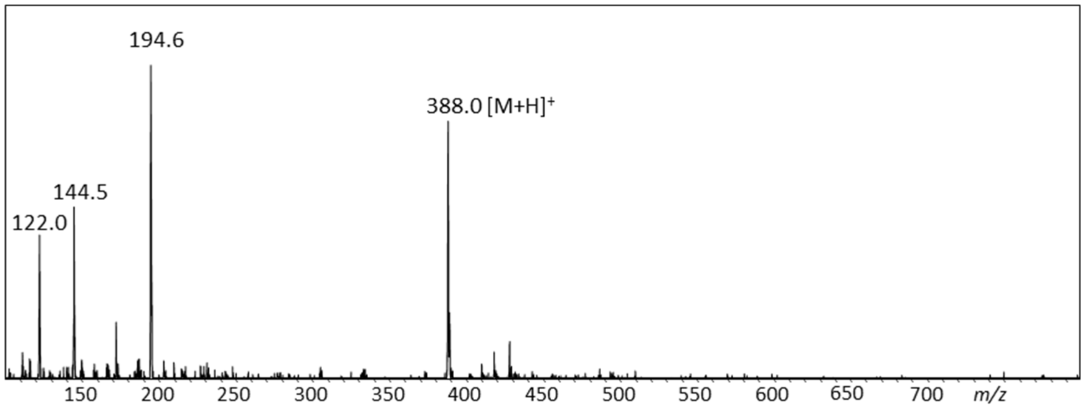

Figure S27. LR-ESI-MS (+ve) spectrum of **DB-N_3**.

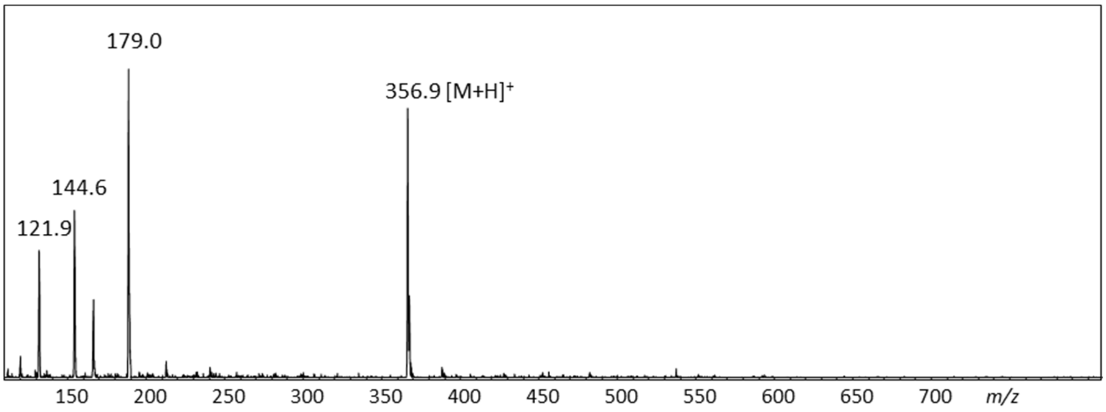

Figure S28. LR-ESI-MS (+ve) spectrum of **DB-alkyne**.

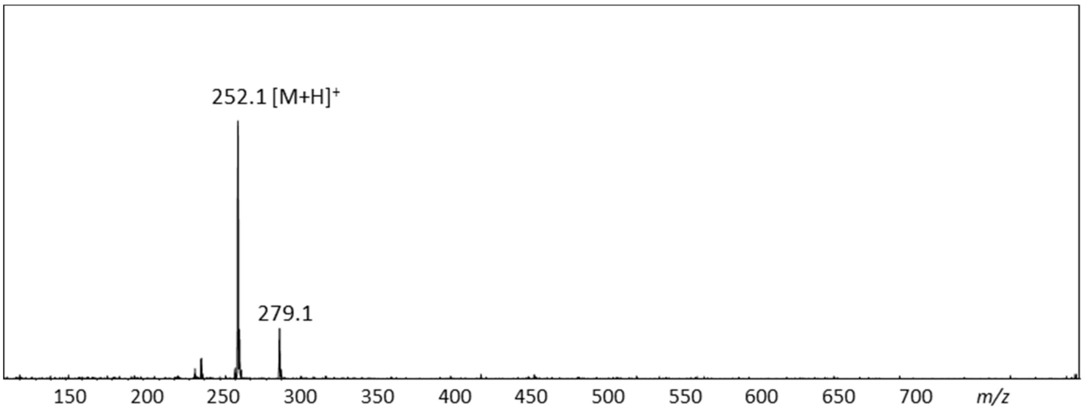

Figure S29. LR-ESI-MS (+ve) spectrum of **Res-alkyne**.

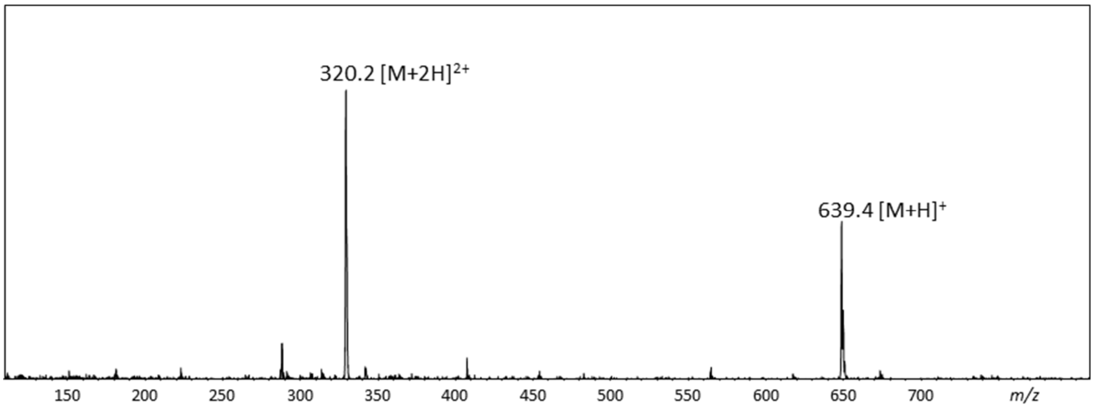

Figure S30. LR-ESI-MS (+ve) spectrum of **DB-Res**.

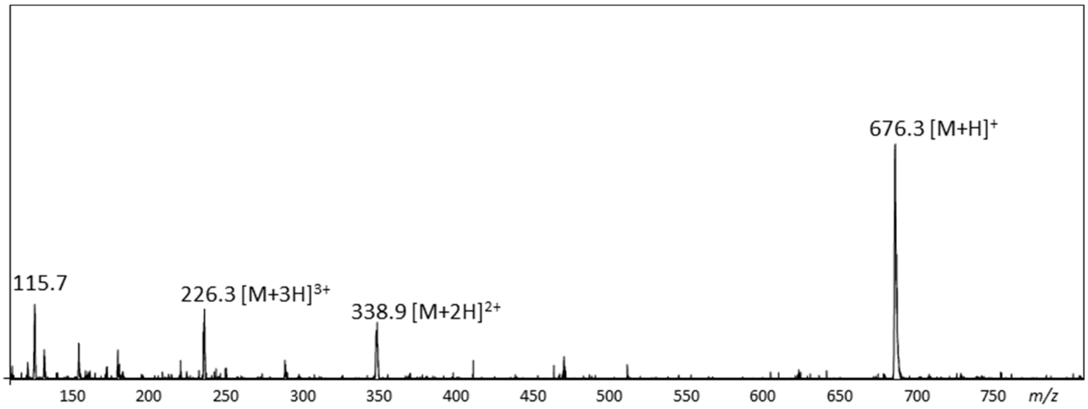

Figure S31. LR-ESI-MS (+ve) spectrum of **DB-Dan**.

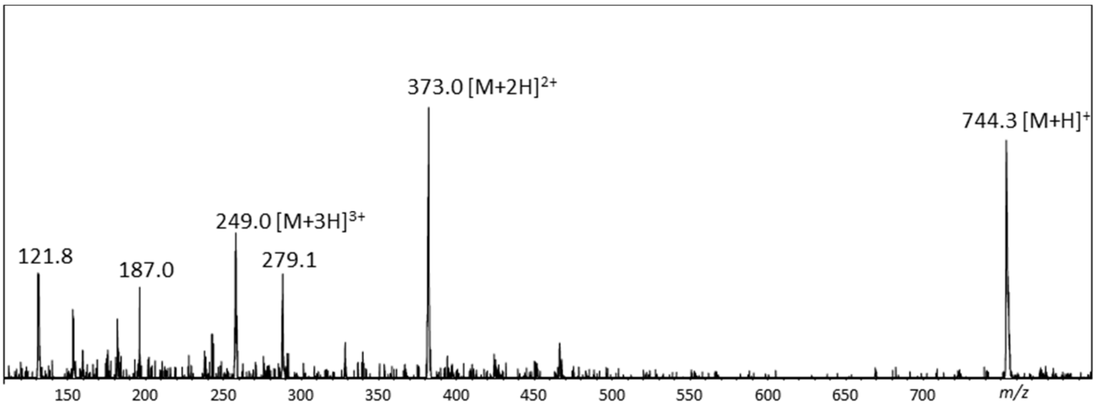

Figure S32. LR-ESI-MS (+ve) spectrum of **DB-TPE**.

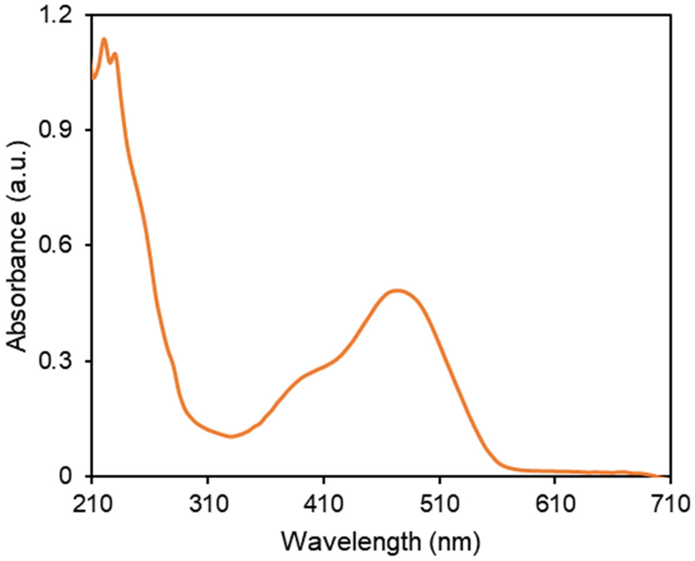

Figure S33. UV-Vis spectrum (1 mM, H_2O, 298 K) of **DB-Res**.

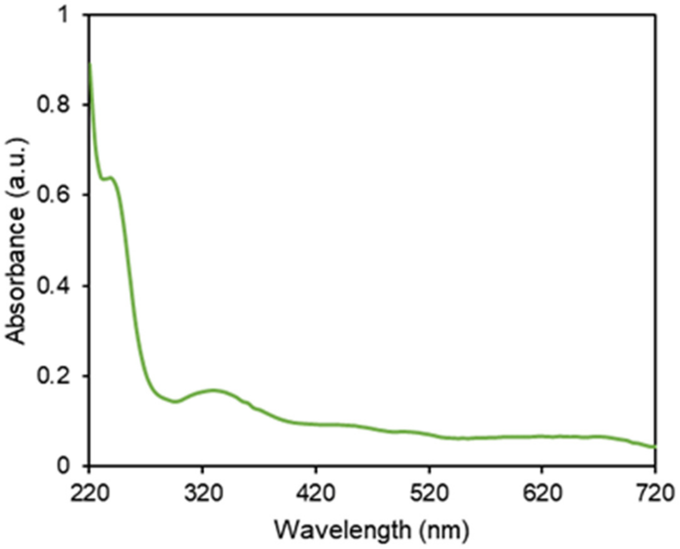

Figure S34. UV-Vis spectrum (50 μM, H_2O, 298 K) of **DB-Dan**.

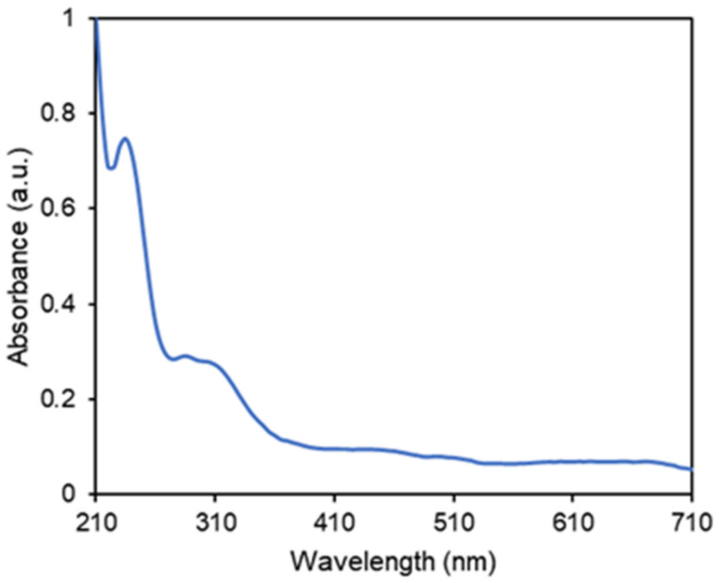

Figure S35. UV-Vis spectrum (20 µM, H_2O, 298 K) of **DB-TPE**.

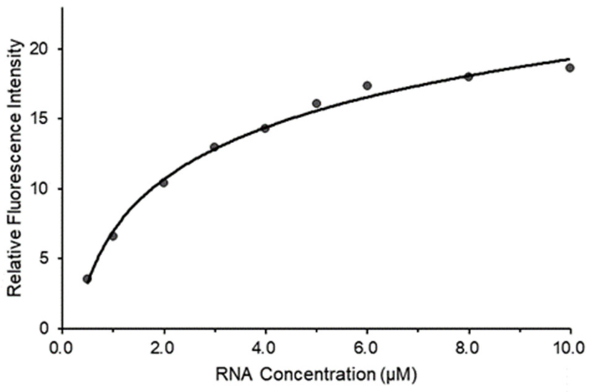

Figure S36. Relative fluorescence intensity at 463 nm of 10 µM **DB-TPE** in the presence of various concentrations of the model $(CAG)_6$ RNA.

References

1. Shi, H.; Kwok, R. T. K.; Liu, J.; Xing, B.; Tang, B. Z.; Liu, B. Real-Time Monitoring of Cell Apoptosis and Drug Screening Using Fluorescent Light-up Probe with Aggregation-Induced Emission Characteristics. *J. Am. Chem. Soc.* **2012**, *134*, 17972–17981.

2. Chen, W.; Mohy Ei Dine, T.; Vincent, S. P. Synthesis of Functionalized Copillar[4+1]Arenes and Rotaxane as Heteromultivalent Scaffolds. *Chem. Commun.* **2021**, *57*, 492–495.
